# Vertical Metabolome Transfer from Mother to Child: An Explainable Machine Learning Method for Detecting Metabolomic Heritability

**DOI:** 10.3390/metabo14030136

**Published:** 2024-02-24

**Authors:** Mario Lovrić, David Horner, Liang Chen, Nicklas Brustad, Ann-Marie Malby Schoos, Jessica Lasky-Su, Bo Chawes, Morten Arendt Rasmussen

**Affiliations:** 1 Copenhagen Prospective Studies on Asthma in Childhood, Herlev and Gentofte Hospital, University of Copenhagen, 2820 Gentofte, Denmark; mario.lovric@inantro.hr (M.L.);; 2 The Lisbon Council, Rue de la Loi 155, 1040 Brussels, Belgium; 3 Centre for Bioanthropology, Institute for Anthropological Research, Gajeva 32, HR-10000 Zagreb, Croatia; 4 Department of Pediatrics, Slagelse Hospital, 4200 Slagelse, Denmark; 5 Department of Medicine, Harvard Medical School, Boston, MA 02115, USA; 6 Channing Division of Network Medicine, Brigham and Women’s Hospital and Harvard Medical School, Boston, MA 02115, USA; 7 Department of Clinical Medicine, Faculty of Health and Medical Sciences, University of Copenhagen, 1172 Copenhagen, Denmark; 8 Department of Food Science, University of Copenhagen, Rolighedsvej 26, 1958 Frederiksberg, Denmark

**Keywords:** PFOS, PFOA, childhood, pregnancy, propensity

## Abstract

Vertical transmission of metabolic constituents from mother to child contributes to the manifestation of disease phenotypes in early life. This study probes the vertical transmission of metabolites from mothers to offspring by utilizing machine learning techniques to differentiate between true mother–child dyads and randomly paired non-dyads. Employing random forests (RF), light gradient boosting machine (LGBM), and logistic regression (Elasticnet) models, we analyzed metabolite concentration discrepancies in mother–child pairs, with maternal plasma sampled at 24 weeks of gestation and children’s plasma at 6 months. The propensity of vertical transfer was quantified, reflecting the likelihood of accurate mother–child matching. Our findings were substantiated against an external test set and further verified through statistical tests, while the models were explained using permutation importance and SHapley Additive exPlanations (SHAP). The best model was achieved using RF, while xenobiotics were shown to be highly relevant in transfer. The study reaffirms the transmission of certain metabolites, such as perfluorooctanoic acid (PFOA), but also reveals additional insights into the maternal influence on the child’s metabolome. We also discuss the multifaceted nature of vertical transfer. These machine learning-driven insights complement conventional epidemiological findings and offer a novel perspective on using machine learning as a methodology for understanding metabolic interactions.

## 1. Introduction

Pregnancy, birth, and the first months of life are crucial in early childhood disease development [1,2]. It is assumed that during pregnancy and early childhood, the mother and child experience a shared exposure to nutrients and environmental intake of chemical compounds (metabolites) [2]. Further, many metabolites are the consequence of enzymatic biochemical conversions stemming from host genomics, or the gut microbiome, which can also shared between mother and child [3]. Metabolomics, through its detailed identification and quantification of biological system metabolites, offers a vital link between metabolic processes and phenotypic outcomes, deepening our insight into the biochemical foundations of physical traits and conditions [4]. Given that metabolites are both products and intermediates in metabolic pathways, their variations serve as early indicators of disease, e.g., potentially predicting clinical symptoms or physiological conditions [5,6]. Furthermore, metabolomics represents a mechanistic information layer for understanding the mode of action for exposures like diet, exercise, and pollutants on disease progression [7]. Understanding which metabolites get passed down from mother to child is crucial in discerning prenatal maternal impacts on the child’s health [2,8,9,10]. For example, per- and poly-fluoroalkyl substances (PFAS), metabolites associated with high rates of vertical transfer [11], have been associated with premature birth [12] and adverse child growth [2] but also show the potential to affect the lipidome [10].

Metabolic transfer from mother to child was investigated in previous work in our group utilizing dried blood spot (DBS) samples at age 2–3 days [13] and using linear correlation analysis. This showed that using dried blood spot (DBS) samples from children aged 2–3 days could identify 11 vertically transferred metabolites as evidence of metabolic transfer from mother to child. One of these metabolites, ergothioneine, has been shown to be a powerful antioxidant and has been suggested to be a plausible therapeutic agent in several diseases [14]. Furthermore, we showed that seven of the vertically transferred metabolites were also found to show persistence in their concentration levels in the child from birth to age six years. Interestingly, many of these vertically transferred metabolites were diet-related and associated with the risk of common infections, eczema, and asthma in early childhood [8]. However, in addition to vertical transfer, as suggested in previous work, there seem to be several routes observed [13]. The literature refers to familial or individual–environmental contributions [15]. Familial variation comprises all heritable and common environmental effects (i.e., arising from genetics or shared environment after conception). While some metabolites are considered purely genetic/heritable and associated with single nucleotide polymorphisms (SNPs) [16,17], systematic research conducted on animals (cattle) has shown that 11 out of 33 researched plasma metabolites could be explained by low to moderate genetic heritability [18] while the other 22 were explained by other factors such as environmental. Hence, both genetics and shared environment play a role. Other means of transfer are suggested, too. Breastfeeding has been researched frequently, showing to be an important route for metabolites from mother to child [1,19,20]. Furthermore, maternal gestational weight gain has been associated with alterations in the infant metabolome [21], pointing out the transfer role during pregnancy. Novel research also presents metabolites as a consequence or a vertical transfer in the gut microbiome, which drives metabolites’ concentration [22]. 

In this study, our objective was to identify metabolites that are transferred from mother to child from pregnancy to 6 months, employing advanced machine learning techniques to expand beyond the limited scope of previous linear models. We propose an approach to model vertical metabolome transfer in a data-driven fashion by utilizing machine learning in an explainable way. We used mother–child pairs from the Copenhagen Prospective Studies on Asthma in Childhood (COPSAC2010) cohort as targets to predict (dependent) variables, which we refer to as “dyads” in this work, a concept also used in genetics [23]. The opposite class is “random dyads,” being pairs of random mother–child observations, i.e., non-families. Hence, our work revolves around the following research questions: (1) Can machine learning and metabolomics be used to detect mother–child dyads? (2) Should non-linearities be taken into account? (3) Are there metabolites which characterize mom–child relationships? Furthermore, we tested whether belonging to a dyad was associated with infections and breastfeeding. 

## 2. Materials and Methods

### 2.1. Study Population

**Figure 1:**
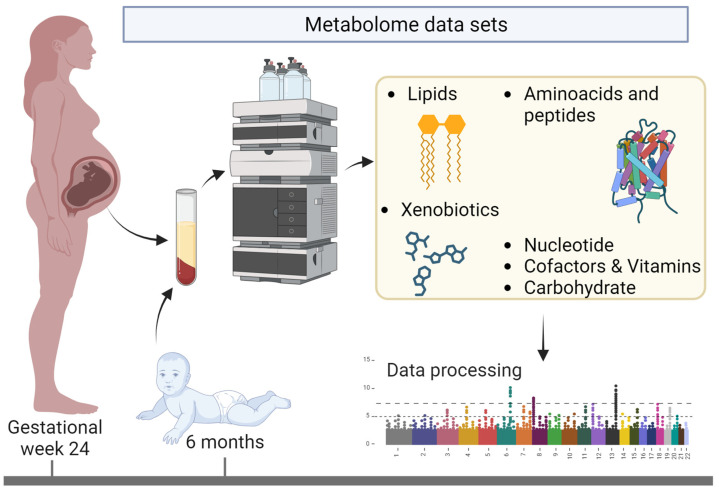
Metabolome sampling time points and chemical categories in the COPSAC2010 cohort selected for this work.

The COPSAC2010 cohort comprised 738 pregnant women and their 700 children, representing a population-based mother–child cohort. These women underwent their first examinations during the 22nd to 26th weeks of pregnancy. The average age of the mothers at the time of their children’s birth was 32.3 ± 4.3 years. The children involved in the study were first examined at the clinic when they were one week old, followed by subsequent visits at ages 1, 3, 6, 12, 18, 24, 30, 36 months, and annually after that until they reached six years, with additional follow-ups at ages 8 and 10. Figure 1 shows the selected timeframe for this study. The full protocol for the cohort, information on withdrawals from the study, and the flowchart are presented in the Supplementary Materials of our previous work [24].

Gestational age was established in the COPSAC2010 study using ultrasonography as part of standard prenatal care. This cohort included infants born preterm and post-term, ranging from 32 to 42 weeks of gestation. In the third trimester, participating women were part of a factorial-designed, double-blind, randomized controlled trial. They were administered either a high dose (2400 IU/day) of vitamin D or placebo on top of the standard pregnancy dose (400 IU/day) of vitamin D [25] and either 2.4 g of n−3 long-chain polyunsaturated fatty acids (LCPUFA), comprising 55% eicosapentaenoic acid (EPA) and 37% docosahexaenoic acid (DHA), or a placebo containing 72% oleic acid and 12% linoleic acid. The study did not include women with endocrine, heart, or kidney diseases or those consuming over 600 IU/day of vitamin D. Information regarding the preparation of samples, the UHPLC-MS/MS analysis, and the measures for quality control was previously published in [1]. The untargeted metabolome from the mother’s and children’s plasma was chemically analyzed by Metabolon, Inc. (Morrisville, NC, USA). This analysis utilized an ACQUITY UHPLC system (Waters, Milford, CT, USA) coupled with a QExactive™ Hybrid Quadrupole-Orbitrap™ mass spectrometer, which features a heated electrospray ionization source (ThermoFisher Scientific, Waltham, MA, USA), operating at a mass resolution of 35,000. The analysis of the processed samples was performed across four distinct platforms: (1) UHPLC-ESI(+)MS/MS tailored to lipophilic substances; (2) UHPLC-ESI(+)MS/MS designed for lipophobic substances; (3) reverse phase UHPLC-ESI(−)MS/MS under basic optimized conditions; (4) HILIC/UHPLC-ESI(−)MS/MS. The identification of metabolites adhered to three specific criteria: retention time/index range, mass accuracy within ±10 ppm, and the MS/MS spectral data. Further details on the analysis were previously published in [13].

### 2.2. Metabolites as Features in the Models

**Figure 2:**
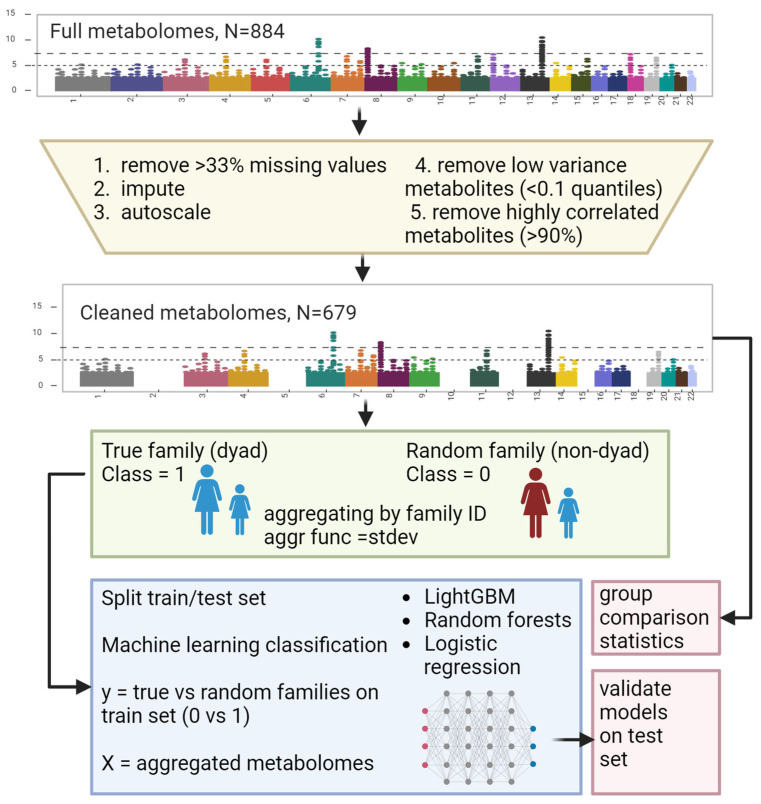
Block diagram of the data preparation procedure (Section 2.2) and machine learning (Section 2.3).

The table features 884 metabolites, many of which are annotated (Tables S1 and S2 together in the Supplement). All data procedures and manipulations were conducted using Python (v3.9.10). The selected timepoints for this analysis were mothers at 24 weeks of pregnancy and children at six months of age, as these are the closest timepoints between mother and child (Figure 1) regarding plasma-based metabolites. Metabolites with over 33% missing values were excluded from the study. Those with fewer missing values were filled in with 1/10 of the smallest concentration for each metabolite, assuming this represents the detection limit. The metabolites were then adjusted to a scale ranging from 0 to 1 (using min–max scaling); another refinement involved discarding the metabolites with the lowest 10% variance. Finally, metabolites with more than 90% Pearson intercorrelation were also removed. The mother–child pairs, i.e., the dyads, were created in an automated Python script by grouping them along the family identification code (ID). At the same time, the aggregation function is the standard deviation of the dyad. Hence, the dyad is represented by its relative positions in the time points metabolomes. An example is provided in the subsequent text. If the mother has a low concentration of metabolite X among the mothers, such as a value of 0.1, and the child has a value of 0.1 amongst the age six months scaled concentrations of metabolite X, the pair will have a standard deviation of 0, and hence they would be related based on metabolite X. The non-pairs or random dyad were created accordingly by shuffling mothers and matching them with random children. This was carried out twice. Hence, there were two times more random dyads in the data set. To balance that out, 30% of the random dyads were dropped from the data, which added additional randomness to the data. The final number of metabolites to enter the study was 679, i.e., standard deviations between mom and child. A schematic of the procedure is presented in Figure 2.

### 2.3. Machine Learning Methods, Statistics, and Model Scoring

The true dyads vs. random dyads stratification was set as a binary classification problem, i.e., a true dyad (mother–child pair) had the label 1 (true). In contrast, random dyads were designated with the value 0 (false). The selected machine learning algorithms were random forest classification (RF) [26], logistic regression with both penalties L1 and L2 (LR), sometimes referred to as Elasticnet [27,28], and LightGBM (LGBM) [29], which all predict a dyad (or not) based on the metabolome being assigned a label of 0 or 1. The algorithms RF and LGBM are ensembles of decision trees and have improved predictive power compared to linear models, such as LR, in many use cases [29,30,31]. We used a procedure presented in our prior work written in the programming language Python. The 10 × cross-validation (CV) was conducted on 80% of data randomly split. The remaining 20% of data represented an external validation set that was not involved in feature selection and model evaluation until the end. Care was taken to split data before [32]. Hyper-parameter optimization was conducted utilizing Bayesian optimization [33,34]. Each model’s hyperparameters are provided in Appendix A. During CV, we also conducted feature selection (Section 2.3). The scoring function and metric for reporting model results is Matthew’s correlation coefficient (MCC) [35,36]. The MCC is equal to the product of the true positives (TP) and true negatives (TN) minus the product of the false positives (FP) and false negatives (FN), all divided by the square root of the product of (TP plus FP), (TP plus FN), (TN plus FP), and (TN plus FN). The choice of metrics is relevant because many metrics do not adequately present the influence of false positives and false negatives. For MCC, the values range between −1 and 1. An MCC above 0.2 should be considered above random or coin-tossing (fair agreement). The final models assessed by their MCC cross-validation scores were then evaluated and reported on the external validation set. 

Statistics were carried out using the t-test on the two groups (dyad vs. not) and adjusting for multiple testing using false discovery rate (FDR) correction with the Benjamini–Hochberg method [37]. 

### 2.4. Feature Selection and Model Explainability

For feature selection in this work, we applied multistage post hoc feature selection [38]. The strategy is based on the importance of permutation for eliminating features [39,40]. Using each of the trained models, the method permutes the values of individual features (one by one) post hoc to assess the relevance of the features concerning the response vector (binary phenotype). The relative decrease in MCC in a pre-trained model caused by a permuted feature is considered a “weight.” The model was trained on the entire feature set in the initial run. Features were then ranked based on their importance, with an average of 5 repeats of permutation per model and run. The average permutation importance was sorted in descending order. Then, the upper 33% of features were selected for the next run while still not involving the external validation set. This was carried out up to seven times with two stopping criteria: (1) CV results did not improve, and (2) a minimum of seven features was reached, which is an arbitrary choice to retain 1% of the feature set (679 metabolites) to ensure a reasonable minimum of features for an ensemble model. This feature selection method was designed to balance selecting the most informative features and avoid the pitfalls of too few or too many features, thereby enhancing the model’s performance and interpretability. Once the models were final, the permutation importance score was used on the final feature set to evaluate the contribution of each feature (metabolite). In addition to using permutation importance, we also used SHAP to understand the models. SHAP (SHapley Additive exPlanations) [41] is a game theory-based approach for explaining the output of ML models. It assigns each feature an importance value for a particular prediction, which is a contribution of each feature to the change in the model output from the baseline prediction. SHAP reveals both global model insights, showing feature importance over the entire model, and local insights for explaining individual predictions [42]. 

## 3. Results

### 3.1. Machine Learning Results 

In assessing the efficacy of our models, we turned to cross-validation of our training data and a subsequent evaluation of the external test set once the final models were set. By comparing the performance of three machine learning models, i.e., random forest (RF), light gradient boosting machine (LGBM), and logistic regression (LR), on a dataset, we observed distinct outcomes. Both RF and LGBM, using seven features, outperformed LR, which used 75 features not reported in detail due to their very high number. All features are shown in Table S3 in the supplement. 

RF was superior in accuracy (0.72) and AUC (0.71) for the test set, closely followed by LGBM (accuracy: 0.66, AUC: 0.68), while LR was surpassed (accuracy: 0.45, AUC: 0.63). Similarly, in terms of the Matthews correlation coefficient (MCC), RF (CV: 0.40, test: 0.42) and LGBM (CV: 0.37, test: 0.36) showed superior performance over LR (CV: 0.12, test: 0.11). The model results are presented in Table 1.

For each of the models, the hyperparameter space is presented in Appendix A, while the final model parameters are shown in Appendix B.

### 3.2. Model Explanations and Important Features

When analyzing the feature importance based on mean permutation importance (PI) for both LGBM and RF models, there was a notable variation in the significance of features between the two (Table 2). 

In LGBM, X-11308 held the highest importance, followed by X-24970 and perfluorooctanoate (PFOA). Other notable features included X-24307, X-12112, X-11372, and X-17653. Contrastingly, in the RF model, although X-11308 also appeared as the most significant feature, it was followed by different features: perfluorooctanoate (PFOA), N6-methyllysine, and X-24970. Less important features in the RF model included N-acetyl-2-aminooctanoate, methionine sulfone, and X-23636. 

**Figure 3:**
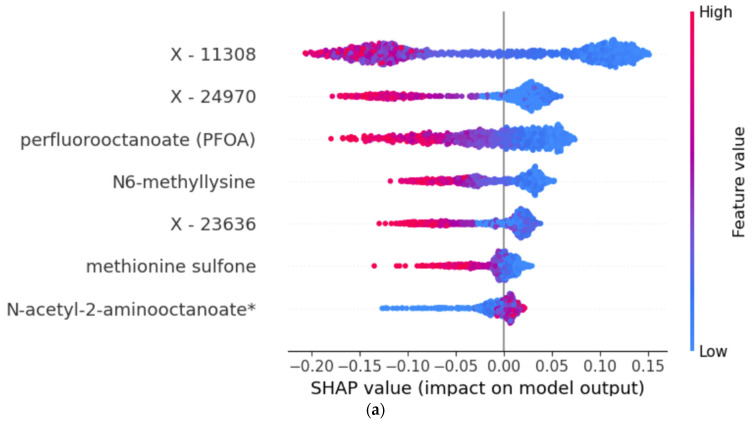

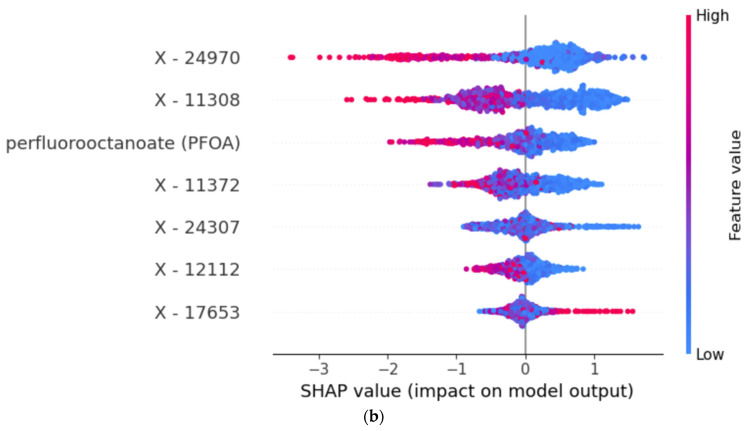
SHAP (SHapley Additive exPlanations) summary plot for the RF model (**a**) and LGBM model (**b**). The two compared groups in post hoc classifier analyses are true (class = 1) and random dyads (class = 0).

In addition to PI, we also applied SHAP to interpret the results. The model interpretations are visualized in Figure 3.

The position on the *x*-axis shows the impact of the feature on the model’s output. Points to the right of the vertical zero line indicate that this feature increases the likelihood of a higher prediction (1, true dyad), while points to the left decrease it (0, random dyad). The features are standard deviations between mom–child pairs; hence, lower values (blue color) mean a lower difference between mom and child. One can observe in the SHAP plot the presence of PFOA in both models. For example, in the RF model and variable PFOA, in Figure 3, one can see that a dyad (1) on the right-hand side of the *x*-axis has a cluster of blue dots (low value) driving the model. This means that a smaller deviation in PFOA concentration between mom and child drives the probability of this belonging to a dyad.

### 3.3. Model Results after Exclusion of Xenobiotics

Another experiment conducted was to test all models with the xenobiotics excluded in the feature sets. Each model was given equal conditions; we merely removed the xenobiotics. The results indicate that for the accuracy on the test set, the RF model achieved the highest score with 0.68, followed by the LGBM at 0.67, and LR lagged behind with a score of 0.55. In terms of the area under the curve (AUC) on the test set, both RF and LGBM models scored equally with 0.68, outperforming LR, which scored 0.59. When considering the MCC for CV, RF had a score of 0.32, which is slightly higher than LGBM’s 0.30 and significantly higher than LR’s 0.07. Finally, for the MCC on the test set, RF again led with 0.39, LGBM was close at 0.37, and LR had a score of 0.26. Overall, RF and LGBM showed similar performance and were notably better than LR across all metrics. While the results are almost reaching those with xenobiotics, the LGBM and LR models suffer from a large number of features (69 each, see Table S4 in the Supplementary Materials), with only the RF model choosing a small number of features: X-11308, 3-carboxy-4-methyl-5-propyl-2-furanpropanoate (CMPF), X-24970, N6, N6-dimethyllysine, bilirubin, sphingomyelin (d18:1/20:1, d18:2/20:0), and 5-methyluridine. This reveals similar observations to full-feature models and statistics. 

### 3.4. Statistical Analysis

**Figure 4:**
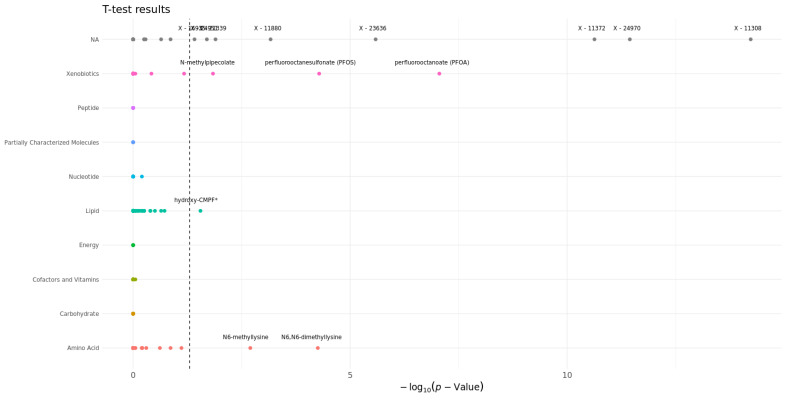
Scatter plot with labeled points (generated using R (version 4.2.2) and ggplot2 [43]) showing metabolites that differentiate the true from the random dyads across biochemical classes. The threshold is set at 0.05. The two compared groups in these statistical tests are true (class = 1) and random dyads (class = 0).

According to the *t*-test results on the two groups, real and random dyads, several metabolites showed significant differences after applying the false discovery rate (FDR) correction using the Benjamini–Hochberg method. Among these, perfluorooctanoate (PFOA) and perfluorooctanesulfonate (PFOS) had particularly low adjusted *p*-values, suggesting strong statistical significance in the analysis. PFOA, with an adjusted *p*-value of approximately 8.81 × 10^−8^, and PFOS, with an adjusted *p*-value of approximately 5.17 × 10^−5^, stood out as the most significantly altered metabolites. N6, N6-dimethyllysine, and N6-methyllysine also showed significant changes, with adjusted *p*-values of approximately 5.55 × 10^−5^ and 1.99 × 10^−3^, respectively. These results suggest noteworthy differences in their levels. However, it is important to note that N-methyl pipecolate and hydroxy-CMPF*, despite having adjusted *p*-values (approximately 1.44 × 10^−2^ and 2.80 × 10^−2^, respectively) that indicate statistical significance, were not as convincingly significant as the other mentioned metabolites. While other metabolites are visible in Figure 4, these are not annotated metabolites; hence, they are not discussed further.

### 3.5. Association to Infections and Breastfeeding

To understand possible associations to health and the relationship to breastfeeding, we ran statistics over the prediction results. The Mann–Whitney U test was applied to assess the differences between groups belonging to a dyad (group 1, N = 23) or not (group 0, N = 85) in relationship to breastfeeding, CRP and infections, provided by the best (RF) model in the test, to generate unbiased results. The results indicated no statistically significant differences with *p*-values as follows: exclusive breastfeeding duration (*p* = 0.721422), infant tonsillitis episodes (*p* = 0.212108), infant gastric episodes (*p* = 0.392920), acute otitis episodes (*p* = 0.578461), CRP at 6 months (*p* = 0.631870), cold episodes (*p* = 0.663752), fever episodes (*p* = 0.699183), pseudocroup episodes (*p* = 0.707612), lower respiratory infection episodes (*p* = 0.718695), trols episodes (*p* = 0.732593), blue spray episodes (*p* = 0.846097), and infection episodes (*p* = 0.846401). Hence, we suggest that no associations exist given this methodology while running it on the test set.

## 4. Discussion

In this study, we explored the relationship between maternal metabolites during pregnancy (24 weeks gestation) and their presence in children at 6 months in the plasma metabolome. We developed a data-driven scale-agnostic procedure using LGBM, RF, and LR to create a new method for detecting metabolomic vertical transfer. The models showed moderate agreement with AUCs at 0.68 for LGBM and 0.71 for RF in both cross-validation and the external test set. The results indicate better prediction reliability and generalization from RF and LGBM compared to LR, with each trained on seven features, suggesting that fewer, more relevant features can lead to more effective and stable models. The variation in feature importance between LGBM and RF models could influence their performance in terms of the MCC, which is a balanced measure of the quality of binary classifications. It also suggests that different features contribute differently to the predictive accuracy of each model. 

Overall, the results in both ML and statistical testing indicate that metabolomics is, to some extent, conserved from mother to child, but also that a fair part of mother–child pairs has no coherent overlap in metabolite concentrations. The predicted variable, dyad vs. random dyads, showed to be a useful strategy, validated by meaningful findings regarding the suggested metabolites. 

Machine learning, particularly ensemble classifiers, has proven to be useful in handling learning processes in complex tasks, here distinguishing between mother–child dyads and random dyads (non-families) in large metabolome [44], microbiome [45], and genetic data sets [46]. Ensemble methods (RF and LGBM), which combine multiple weak learners [26] to obtain better predictive performance, inherently manage the high dimensionality and heterogeneity of metabolomic data more effectively. They can capture complex, non-linear relationships between variables that are often missed by statistical models, such as smaller phenotypes represented by regions in the metabolome feature space. Moreover, ensemble classifiers offer robustness against overfitting, improved accuracy through aggregating predictions, and the ability to handle missing values and unbalanced data, while frequentist statistics often struggle with these issues. Frequentist methods typically rely on assumptions about the distribution of each metabolite concentration and independence from each other, which may not hold true in complex metabolomics datasets. They may not effectively deal with high-dimensional spaces or capture the intricate relationships within the data, leading to potential biases or inaccuracies. Additionally, frequentist approaches demand hypotheses, while here, the process was rather data-driven. 

Regarding model explainability and selected metabolites, our results demonstrate a consistent vertical transfer of PFOA from mothers to children, as evidenced by its presence in two distinct models and supported by statistical analysis. PFOA, absorbed primarily through contaminated food and water and, to a lesser extent, through polluted air, exhibit a lipophilic nature, distributing throughout the body with a notable affinity for accumulating in fatty tissues [47]. The slow elimination of PFOA poses challenges, contributing to their persistence and potential long-term health implications, as evidenced by associations with intrauterine growth and asthma [2]. These findings underscore the need for continued research and regulatory scrutiny to address the complexities of xenobiotic exposure during critical developmental stages and suggest a possible path through breastfeeding [19]. In addition to PFOA, our study unveiled the vertical transfer of other metabolites, N6-methyllysine, N6, N6-dimethyllysine, and N-acetyl-2-aminooctanoate, during the intra-uterine period. N6-methyllysine, involved in various cellular processes, and N-acetyl-2-aminooctanoate, with potential metabolic and developmental significance, add depth (by means of non-linear model results) to our understanding of the molecular interplay between maternal and child metabolic profiles. We previously found vertical transfer by means of N6-methyllysine on DBS samples [13]. Research has suggested that this metabolite is related to genetics [48], which is also valid for N6, N6-dimethyllysine [49], which are deemed to be markers for intrapersonal biomarker stability [49]. 

Given the importance of vertical transfer on child health outcomes, our study adds to the literature by providing non-linear machine-learning driven methodologies for analyzing these data, which may capture this complex biology better [50]. Childhood is a sensitive period of development, and our findings are substantiated by identifying metabolites which have been linked with relevant health outcomes in offspring. 

Our study further confirms previous findings regarding the transfer of hydroxy-CMPF, a metabolite associated with fatty fish intake and fish oil supplementation in pregnancy [1]. The fish oil intervention, which was conducted in this cohort, adds a unique dimension, implicating dietary choices in the maternal–fetal transfer of specific metabolites. Understanding the dynamics of hydroxy–CMPF transfer may contribute valuable insights into the broader context of nutritional interventions and genetic predispositions, underscoring the multifaceted nature of maternal–fetal metabolic interactions and their potential implications for early childhood development. 

While it is assumed that vertical transfer is the mechanism, one can assume that shared environmental exposure and/or nutrition can play a role, too. In our previous work, we discussed the concept of microbiome transfer from mom to child [3]. We introduced the idea of persistent and transient transfer, e.g., through, vaginal delivery. The observation was that the microbiota is transferred directly, with one part being persistent and the other transient. We assume here that this could be the case for the metabolome, too. Moreover, knowing that microbiota can drive the metabolome [51], we suggest that in such a vast space of metabolites and given current research, we might have a hard time factorizing contribution to mom–child metabolome transfers when comparing genetic heritability, shared environment, breastfeeding, and microbiome transfer. In recent research by [52], it was suggested that exposure to xenobiotics during pregnancy could also be transferred to offspring, either through the placenta or maternal milk and cause subsequent disease. Further research by the same group also revealed the effects of maternal exposure to xenobiotics that can induce changes in the microbiome [9] and that xenobiotic exposure mediated through the mother’s diet and breastfeeding can affect the mother’s milk lipidome [10]. When comparing the research of the Orešič group [9,10,52] with our research on microbiome transfer [3], we can derive that exposure to xenobiotics can not only be transferred directly via the placenta and breastfeeding but can also have an interplay with the microbiome. This causes changes in the lipidome, and we hypothesize that this could trigger changes in the metabolome of offspring in general. Hence, offspring at 6 months can still receive a large contribution from different pathways beyond vertical transfer, while vertical transfer could happen directly or mediated through the microbiome.

### Limitations and Future Research

Overall, our study, using machine learning models, demonstrated that several metabolites are transferred from pregnancy to six months in children. Such associations can provide suggestions for the prevention of early childhood diseases, not only in early life but also during pregnancy. Several metabolites in the data set are still not annotated and will present interesting findings in the future. More work is needed to understand the previously listed factors in metabolome transfer and understand all possible routes. The literature suggests several routes regarding the metabolome, such as placental transfer during pregnancy, shared nutrition and environment, and hereditability by means of genetics and microbiome contribution. In the long lists of analyzed metabolites, each needs to be solved case by case to understand the contributions. Another set of limitations is related to the computational techniques used. Even though the model results show good performance, they are still far from accurate concerning classification. Hence, we can only explain a part of the cohort, while the models fail for some. This suggests that there are missing data to understand dyads vs. random dyads or that the method of choosing the dyad needs another approach. In our future research, we aim to obtain additional data on the unknown metabolites, improve the models, and explore longer periods in the children’s lives.

## 5. Conclusions

In this work, we developed a new methodology for inspecting vertical metabolome transfer from mother to child based on machine learning. The features used were the differences in mother–child metabolite concentration in the COPSAC2010 cohort on 679 metabolites in a final data set. The machine learning methods we tested were LR (elasticnet), RF, and LGBM. We showed that RF was the dominant algorithm for this task, while LR achieved the worst results. A subsequent feature analysis by means of permutation importance and SHAP showed that PFOA dominated the models as the most relevant feature, which was also confirmed by statistical tests. In addition to PFOA, statistics also showed the importance of PFOS. Both being xenobiotics, this shows how vertical transfer is affected by environmental exposure.

## Figures and Tables

**Table 1:** Model results.

	RF	LGBM	LR
Accuracy Test set	0.72	0.66	0.45
AUC Test set	0.71	0.68	0.63
MCC CV	0.40	0.37	0.12
MCC Test set	0.42	0.36	0.11

**Table 2:** Permutation importance (PI) in the final models LGBM and RF.

	LGBM Feature	LGBM PI	RF Feature	RF PI
1.	X-11308	0.202	X-11308	0.106
2.	X-24970	0.191	perfluorooctanoate (PFOA)	0.036
3.	perfluorooctanoate (PFOA)	0.119	N6-methyllysine	0.035
4.	X-24307	0.104	X-24970	0.030
5.	X-12112	0.090	N-acetyl-2-aminooctanoate	0.021
6.	X-11372	0.081	methionine sulfone	0.013
7.	X-17653	0.062	X-23636	0.003

## Data Availability

All data that support the findings in this study, including clinical data, are available from the corresponding author upon reasonable request: participant-level personally identifiable data are protected under the Danish Data Protection Act and European Regulation 2016/679 of the European Parliament and of the Council (GDPR) that prohibit distribution even in pseudo-anonymized form, but can be made available under a data transfer agreement as a collaboration effort.

## Supplementary Materials

The following supporting information can be downloaded at https://www.mdpi.com/article/10.3390/metabo14030136/s1, Table S1: List of metabolites which entered the models (N = 679); Table S2: The list of 205 metabolites out of the 884, which didn’t enter the models; Table S3: Metabolites chosen in each of the final machine learning models; Table S4: Metabolites chosen in each of the final machine learning models without xenobiotics.

## Appendix A. Model Hyperparameter Space

(1) Random Forest

The maximum depth of the trees was set between 5 and 10. The number of trees in the forest (n_estimators) was varied between 200 and 300. The max_samples parameter, defining the fraction of the total number of samples to draw from the original dataset to train each base estimator, was set between 0.05 and 0.15. The minimum number of samples required to split an internal node (min_samples_split) ranged from 5 to 15. Lastly, the class_weight_ratio, influencing the weight of classes in the model, was adjusted between 1 and 5.

(2) Logistic Regression

For this model, the l1_ratio, determining the mix of L1 and L2 regularization, varied logarithmically between 10^(−4) and 10^(0). The regularization strength (C) was also adjusted logarithmically between 10^(−2) and 10^(0). The class_weight_ratio was set in a range from 1 to 5, influencing the importance of classes in the model.

(3) LightGBM (LGBM)

The light gradient boosting machine was tuned with the following parameters: the num_leaves, specifying the number of leaves in the tree, was set between 10 and 50. The learning rate, crucial for the rate of model learning, ranged from 0.01 to 0.1. The number of boosting iterations (n_estimators) was between 200 and 500. The class_weight_ratio, similar to the other models, was set between 1 and 5. Additionally, the minimum number of data points (min_child_samples) required in a leaf node was adjusted from 3 to 8.

## Appendix B. Final Model Hyperparameters

(1) Random Forest

“params”: {“bootstrap”: true, “max_depth”: 5, “n_estimators”: 270, “max_samples”: 0.1366176145774935, “min_samples_split”: 11, “class_weight”: {“0”: 1, “1”: 1.6239780813448106}, “random_state”: 42

(2) Logistic Regression

“params”: {“penalty”: “elasticnet”, “C”: 1.0, “solver”: “saga”, “l1_ratio”: 1.0, “class_weight”: {“0”: 1, “1”: 7.009537649330668}, “tol”: 0.01, “random_state”: 42

(3) LightGBM (LGBM)

“params”: {“num_leaves”: 35, “learning_rate”: 0.01, “n_estimators”: 384, “min_child_samples”: 8, “class_weight”: {“0”: 1, “1”: 3.0377935914367953}, “random_state”: 42
