# Supplementary material for: Vertical Metabolome Transfer from Mother to Child: An Explainable Machine Learning Method for Detecting Metabolomic Heritability
Source: Metabolites. 2024 Feb 24;14(3):136. doi: 10.3390/metabo14030136 (PMC10972480; doi:10.3390/metabo14030136)
Supplement: Supplementary file 1 [file metabolites-14-00136-s001.zip › metabolites-2816191-supplementary.pdf]

Supplementary materials

Vertical metabolome transfer from mother to child: an explainable machine learning method for detecting metabolomic heritability

Mario Lovric, David Horner, Liang Chen, Nicklas Brustad, Ann-Marie Malby Schoos, Jessica Lasky-Su, Bo Chawes, Morten Arendt Rasmussen

Table S1. List of metabolites which entered the models (N=679).

	Column1	Column2	Column3	Column4
0	linoleate (18:2n6)_Long Chain Polyunsaturated Fatty Acid (n3 and n6)_Lipid	allantoin_Purine Metabolism, (Hypo)Xanthine/Inosine containing_Nucleotide	arachidonate (20:4n6)_Long Chain Polyunsaturated Fatty Acid (n3 and n6)_Lipid	arachidate (20:0)_Long Chain Saturated Fatty Acid_Lipid
1	myo-inositol_Inositol Metabolism_Lipid	isoleucine_Leucine, Isoleucine and Valine Metabolism_Amino Acid	alanine_Alanine and Aspartate Metabolism_Amino Acid	3-methoxytyrosine_Tyrosine Metabolism_Amino Acid
2	beta-hydroxyisovalerate_Leucine, Isoleucine and Valine Metabolism_Amino Acid	threonine_Glycine, Serine and Threonine Metabolism_Amino Acid	tyrosine_Tyrosine Metabolism_Amino Acid	lysine_Lysine Metabolism_Amino Acid
3	methionine_Methionine, Cysteine, SAM and Taurine Metabolism_Amino Acid	malate_TCA Cycle_Energy	nonadecanoate (19:0)_Long Chain Saturated Fatty Acid_Lipid	stearate (18:0)_Long Chain Saturated Fatty Acid_Lipid
4	pentadecanoate (15:0)_Long Chain Saturated Fatty Acid_Lipid	myristate (14:0)_Long Chain Saturated Fatty Acid_Lipid	kynurenate_Tryptophan Metabolism_Amino Acid	5,6-dihydrothymine_Pyrimidine Metabolism, Thymine containing_Nucleotide
5	5-methylthioadenosine (MTA)_Polyamine Metabolism_Amino Acid	succinate_TCA Cycle_Energy	ornithine_Urea cycle; Arginine and Proline Metabolism_Amino Acid	5-oxoproline_Glutathione Metabolism_Amino Acid
6	N6,N6,N6-trimethyllysine_Lysine Metabolism_Amino Acid	pantothenate (Vitamin B5)_Pantothenate and CoA Metabolism_Cofactors and Vitamins	picolinate_Tryptophan Metabolism_Amino Acid	glycerol_Glycerolipid Metabolism_Lipid
7	kynurenine_Tryptophan Metabolism_Amino Acid	sarcosine_Glycine, Serine and Threonine Metabolism_Amino Acid	tartarate_Food Component/Plant_Xenobiotics	glucuronate_Aminosugar Metabolism_Carbohydrate
8	3-hydroxyisobutyrate_Leucine, Isoleucine and Valine Metabolism_Amino Acid	carnitine_Carnitine Metabolism_Lipid	choline_Phospholipid Metabolism_Lipid	4-acetamidobutanoate_Polyamine Metabolism_Amino Acid
9	alpha-tocopherol_Tocopherol Metabolism_Cofactors and Vitamins	chenodeoxycholate_Primary Bile Acid Metabolism_Lipid	citrate_TCA Cycle_Energy	1-methyladenosine_Purine Metabolism, Adenine containing_Nucleotide
10	3-aminoisobutyrate_Pyrimidine Metabolism, Thymine containing_Nucleotide	vanillylmandelate (VMA)_Tyrosine Metabolism_Amino Acid	3-methyl-2-oxovalerate_Leucine, Isoleucine and Valine Metabolism_Amino Acid	3-methylhistidine_Histidine Metabolism_Amino Acid
11	xanthurenate_Tryptophan Metabolism_Amino Acid	4-guanidinobutanoate_Guanidino and Acetamido Metabolism_Amino Acid	5-hydroxylysine_Lysine Metabolism_Amino Acid	imidazole lactate_Histidine Metabolism_Amino Acid
12	glycerate_Glycolysis, Gluconeogenesis, and Pyruvate Metabolism_Carbohydrate	N-acetylglutamate_Glutamate Metabolism_Amino Acid	methylsuccinate_Leucine, Isoleucine and Valine Metabolism_Amino Acid	3-phenylpropionate (hydrocinnamate)_Benzoate Metabolism_Xenobiotics

13	hippurate_Benzoate Metabolism_Xenobiotics	ethylmalonate_Leucine, Isoleucine and Valine Metabolism_Amino Acid	ribitol_Pentose Metabolism_Carbohydrate	N-acetylalanine_Alanine and Aspartate Metabolism_Amino Acid
14	glycerophosphorylcholine (GPC)_Phospholipid Metabolism_Lipid	phosphoethanolamine (PE)_Phospholipid Metabolism_Lipid	urate_Purine Metabolism, (Hypo)Xanthine/Inosine containing_Nucleotide	arginine_Urea cycle; Arginine and Proline Metabolism_Amino Acid
15	caprate (10:0)_Medium Chain Fatty Acid_Lipid	fumarate_TCA Cycle_Energy	heptanoate (7:0)_Medium Chain Fatty Acid_Lipid	serine_Glycine, Serine and Threonine Metabolism_Amino Acid
16	valine_Leucine, Isoleucine and Valine Metabolism_Amino Acid	4-hydroxyphenylpyruvate_Tyrosine Metabolism_Amino Acid	urea_Urea cycle; Arginine and Proline Metabolism_Amino Acid	cortisol_Corticosteroids_Lipid
17	cortisone_Corticosteroids_Lipid	sphingosine_Sphingosines_Lipid	dihomolinoleate (20:2n6)_Long Chain Polyunsaturated Fatty Acid (n3 and n6)_Lipid	2-hydroxystearate_Fatty Acid, Monohydroxy_Lipid
18	retinol (Vitamin A)_Vitamin A Metabolism_Cofactors and Vitamins	gamma-glutamylhistidine_Gamma-glutamyl Amino Acid_Peptide	quinate_Food Component/Plant_Xenobiotics	indolelactate_Tryptophan Metabolism_Amino Acid
19	azelate (C9-DC)_Fatty Acid, Dicarboxylate_Lipid	cys-gly, oxidized_Glutathione Metabolism_Amino Acid	gamma-glutamylleucine_Gamma-glutamyl Amino Acid_Peptide	methionine sulfoxide_Methionine, Cysteine, SAM and Taurine Metabolism_Amino Acid
20	theobromine_Xanthine Metabolism_Xenobiotics	theophylline_Xanthine Metabolism_Xenobiotics	eicosapentaenoate (EPA; 20:5n3)_Long Chain Polyunsaturated Fatty Acid (n3 and n6)_Lipid	glycocholate_Primary Bile Acid Metabolism_Lipid
21	taurochenodeoxycholate_Primary Bile Acid Metabolism_Lipid	taurocholate_Primary Bile Acid Metabolism_Lipid	cysteine_Methionine, Cysteine, SAM and Taurine Metabolism_Amino Acid	proline_Urea cycle; Arginine and Proline Metabolism_Amino Acid
22	quinolinate_Nicotinate and Nicotinamide Metabolism_Cofactors and Vitamins	1,2-dipalmitoyl-GPC (16:0/16:0)_Phosphatidylcholine (PC)_Lipid	1-myristoyl-2-palmitoyl-GPC (14:0/16:0)_Phosphatidylcholine (PC)_Lipid	1-palmitoyl-2-oleoyl-GPE (16:0/18:1)_Phosphatidylethanolamine (PE)_Lipid
23	1-stearoyl-2-oleoyl-GPS (18:0/18:1)_Phosphatidylserine (PS)_Lipid	1-stearoyl-GPI (18:0)_Lysophospholipid_Lipid	stearoyl sphingomyelin (d18:1/18:0)_Sphingomyelins_Lipid	1,5-anhydroglucitol (1,5-AG)_Glycolysis, Gluconeogenesis, and Pyruvate Metabolism_Carbohydrate
24	maleate_Fatty Acid, Dicarboxylate_Lipid	tartronate (hydroxymalonate)_Food Component/Plant_Xenobiotics	oxalate (ethanedioate)_Ascorbate and Aldarate Metabolism_Cofactors and Vitamins	iminodiacetate (IDA)_Chemical_Xenobiotics
25	2-oleoylglycerol (18:1)_Monoacylglycerol_Lipid	taurine_Methionine, Cysteine, SAM and Taurine Metabolism_Amino Acid	citrulline_Urea cycle; Arginine and Proline Metabolism_Amino Acid	3-hydroxyoctanoate_Fatty Acid, Monohydroxy_Lipid
26	3-hydroxydecanoate_Fatty Acid, Monohydroxy_Lipid	4-methyl-2-oxopentanoate_Leucine, Isoleucine and Valine Metabolism_Amino Acid	phenyllactate (PLA)_Phenylalanine Metabolism_Amino Acid	alpha-hydroxyisocaproate_Leucine, Isoleucine and Valine Metabolism_Amino Acid
27	homoarginine_Urea cycle; Arginine and Proline Metabolism_Amino Acid	homocitrulline_Urea cycle; Arginine and Proline Metabolism_Amino Acid	EDTA_Chemical_Xenobiotics	cysteine s-sulfate_Methionine, Cysteine, SAM and Taurine Metabolism_Amino Acid
28	gamma-glutamylglutamine_Gamma-glutamyl Amino Acid_Peptide	gamma-glutamyltyrosine_Gamma-glutamyl Amino Acid_Peptide	indoleacetate_Tryptophan Metabolism_Amino Acid	thyroxine_Tyrosine Metabolism_Amino Acid
29	1-methylnicotinamide_Nicotinate and Nicotinamide Metabolism_Cofactors and Vitamins	3-indoxyl sulfate_Tryptophan Metabolism_Amino Acid	N-acetylglycine_Glycine, Serine and Threonine Metabolism_Amino Acid	creatine_Creatine Metabolism_Amino Acid

30	galactonate_Fructose, Mannose and Galactose Metabolism_Carbohydrate	ribonate_Pentose Metabolism_Carbohydrate	threonate_Ascorbate and Aldarate Metabolism_Cofactors and Vitamins	N-formylmethionine_Methionine, Cysteine, SAM and Taurine Metabolism_Amino Acid
31	hypoxanthine_Purine Metabolism, (Hypo)Xanthine/Inosine containing_Nucleotide	betaine_Glycine, Serine and Threonine Metabolism_Amino Acid	xanthine_Purine Metabolism, (Hypo)Xanthine/Inosine containing_Nucleotide	N-(2-furoyl)glycine_Food Component/Plant_Xenobiotics
32	3-ureidopropionate_Pyrimidine Metabolism, Uracil containing_Nucleotide	androsterone sulfate_Androgenic Steroids_Lipid	3-carboxy-4-methyl-5-propyl-2-furanpropanoate (CMPF)_Fatty Acid, Dicarboxylate_Lipid	3-(4-hydroxyphenyl)lactate (HPLA)_Tyrosine Metabolism_Amino Acid
33	acetylcarnitine (C2)_Fatty Acid Metabolism (Acyl Carnitine, Short Chain)_Lipid	hydroxyproline_Urea cycle; Arginine and Proline Metabolism_Amino Acid	hexanoylcarnitine (C6)_Fatty Acid Metabolism (Acyl Carnitine, Medium Chain)_Lipid	glycochenodeoxycholate_Primary Bile Acid Metabolism_Lipid
34	4-imidazoleacetate_Histidine Metabolism_Amino Acid	1-methyl-4-imidazoleacetate_Histidine Metabolism_Amino Acid	N-acetylneuraminate_Aminosugar Metabolism_Carbohydrate	dodecanedioate (C12)_Fatty Acid, Dicarboxylate_Lipid
35	3-hydroxy-2-ethylpropionate_Leucine, Isoleucine and Valine Metabolism_Amino Acid	trigonelline (N'-methylnicotinate)_Nicotinate and Nicotinamide Metabolism_Cofactors and Vitamins	indolepropionate_Tryptophan Metabolism_Amino Acid	docosadienoate (22:2n6)_Long Chain Polyunsaturated Fatty Acid (n3 and n6)_Lipid
36	myristoleate (14:1n5)_Long Chain Monounsaturated Fatty Acid_Lipid	dehydroepiandrosterone sulfate (DHEA-S)_Androgenic Steroids_Lipid	propionylcarnitine (C3)_Fatty Acid Metabolism (also BCAA Metabolism)_Lipid	3-hydroxylaurate_Fatty Acid, Monohydroxy_Lipid
37	caproate (6:0)_Medium Chain Fatty Acid_Lipid	10-undecenoate (11:1n1)_Medium Chain Fatty Acid_Lipid	docosapentaenoate (DPA; 22:5n3)_Long Chain Polyunsaturated Fatty Acid (n3 and n6)_Lipid	2-linoleoylglycerol (18:2)_Monoacylglycerol_Lipid
38	phenol sulfate_Tyrosine Metabolism_Amino Acid	pregnen-diol disulfate*_Pregnenolone Steroids_Lipid	X - 11261_NA_NA	bilirubin (E,E)*_Hemoglobin and Porphyrin Metabolism_Cofactors and Vitamins
39	glycocholenate sulfate*_Secondary Bile Acid Metabolism_Lipid	pregnenediol sulfate (C21H34O5S)*_Pregnenolone Steroids_Lipid	glycolithocholate sulfate*_Secondary Bile Acid Metabolism_Lipid	taurocholenate sulfate*_Secondary Bile Acid Metabolism_Lipid
40	andro steroid monosulfate C19H28O6S (1)*_Androgenic Steroids_Lipid	adrenate (22:4n6)_Long Chain Polyunsaturated Fatty Acid (n3 and n6)_Lipid	X - 11787_NA_NA	X - 11795_NA_NA
41	1-arachidonoyl-GPC* (20:4)*_Lysophospholipid_Lipid	1-palmitoleoyl-GPC* (16:1)*_Lysophospholipid_Lipid	gamma-glutamylthreonine_Gamma-glutamyl Amino Acid_Peptide	gamma-glutamylphenylalanine_Gamma-glutamyl Amino Acid_Peptide
42	isobutyrylcarnitine (C4)_Leucine, Isoleucine and Valine Metabolism_Amino Acid	pseudouridine_Pyrimidine Metabolism, Uracil containing_Nucleotide	palmitoleate (16:1n7)_Long Chain Monounsaturated Fatty Acid_Lipid	eicosenoate (20:1n9 or 1n11)_Long Chain Monounsaturated Fatty Acid_Lipid
43	gamma-glutamyl-epsilon-lysine_Gamma-glutamyl Amino Acid_Peptide	piperine_Food Component/Plant_Xenobiotics	N-acetylthreonine_Glycine, Serine and Threonine Metabolism_Amino Acid	decanoylcarnitine (C10)_Fatty Acid Metabolism (Acyl Carnitine, Medium Chain)_Lipid
44	N-acetylglutamine_Glutamate Metabolism_Amino Acid	N-acetylhistidine_Histidine Metabolism_Amino Acid	gamma-glutamyltryptophan_Gamma-glutamyl Amino Acid_Peptide	gamma-glutamylglycine_Gamma-glutamyl Amino Acid_Peptide
45	N-acetylphenylalanine_Phenylalanine Metabolism_Amino Acid	myristoylcarnitine (C14)_Fatty Acid Metabolism (Acyl Carnitine, Long Chain Saturated)_Lipid	N-acetylarginine_Urea cycle; Arginine and Proline Metabolism_Amino Acid	1-palmitoyl-GPC (16:0)_Lysophospholipid_Lipid

46	N-acetyltryptophan_Tryptophan Metabolism_Amino Acid	1-stearoyl-GPC (18:0)_Lysophospholipid_Lipid	10-nonadecenoate (19:1n9)_Long Chain Monounsaturated Fatty Acid_Lipid	campesterol_Sterol_Lipid
47	hyocholate_Secondary Bile Acid Metabolism_Lipid	1-arachidonoyl-GPI* (20:4)*_Lysophospholipid_Lipid	stachydrine_Food Component/Plant_Xenobiotics	phosphocholine_Phospholipid Metabolism_Lipid
48	isovalerylcarnitine (C5)_Leucine, Isoleucine and Valine Metabolism_Amino Acid	stearoylcarnitine (C18)_Fatty Acid Metabolism (Acyl Carnitine, Long Chain Saturated)_Lipid	1-linoleoyl-GPC (18:2)_Lysophospholipid_Lipid	1-palmitoyl-GPA (16:0)_Lysophospholipid_Lipid
49	sphingosine 1-phosphate_Sphingosines_Lipid	laurylcarnitine (C12)_Fatty Acid Metabolism (Acyl Carnitine, Medium Chain)_Lipid	isovalerylglycine_Leucine, Isoleucine and Valine Metabolism_Amino Acid	7-methylguanine_Purine Metabolism, Guanine containing_Nucleotide
50	phenylacetylglutamine_Acetylated Peptides_Peptide	prolylhydroxyproline_Urea cycle; Arginine and Proline Metabolism_Amino Acid	N4-acetylcytidine_Pyrimidine Metabolism, Cytidine containing_Nucleotide	5-methyluridine (ribothymidine)_Pyrimidine Metabolism, Uracil containing_Nucleotide
51	N2,N2-dimethylguanosine_Purine Metabolism, Guanine containing_Nucleotide	N6-carbamoylthreonyladenosine_Purine Metabolism, Adenine containing_Nucleotide	oleoylcarnitine (C18)_Fatty Acid Metabolism (Acyl Carnitine, Monounsaturated)_Lipid	1-arachidonoyl-GPE (20:4n6)*_Lysophospholipid_Lipid
52	2-palmitoyl-GPC* (16:0)*_Lysophospholipid_Lipid	1-palmitoyl-GPI* (16:0)_Lysophospholipid_Lipid	catechol sulfate_Benzoate Metabolism_Xenobiotics	tiglyl carnitine (C5)_Leucine, Isoleucine and Valine Metabolism_Amino Acid
53	isobutyrylglycine (C4)_Leucine, Isoleucine and Valine Metabolism_Amino Acid	4-hydroxyhippurate_Benzoate Metabolism_Xenobiotics	1-oleoyl-GPE (18:1)_Lysophospholipid_Lipid	1-palmitoyl-GPE (16:0)_Lysophospholipid_Lipid
54	N-acetyl-aspartyl-glutamate (NAAG)_Glutamate Metabolism_Amino Acid	tetradecanedioate (C14)_Fatty Acid, Dicarboxylate_Lipid	hexadecanedioate (C16)_Fatty Acid, Dicarboxylate_Lipid	dihomolinolenate (20:3n3 or 3n6)_Long Chain Polyunsaturated Fatty Acid (n3 and n6)_Lipid
55	thymol sulfate_Food Component/Plant_Xenobiotics	4-vinylphenol sulfate_Benzoate Metabolism_Xenobiotics	4-ethylphenyl sulfate_Benzoate Metabolism_Xenobiotics	p-cresol sulfate_Benzoate Metabolism_Xenobiotics
56	1-linoleoyl-GPI* (18:2)*_Lysophospholipid_Lipid	1-linoleoyl-GPE (18:2)*_Lysophospholipid_Lipid	1-oleoyl-GPI (18:1)_Lysophospholipid_Lipid	gamma-glutamylglutamate_Gamma-glutamyl Amino Acid_Peptide
57	2-hydroxy-3-methylvalerate_Leucine, Isoleucine and Valine Metabolism_Amino Acid	deoxycarnitine_Carnitine Metabolism_Lipid	N6-acetyllysine_Lysine Metabolism_Amino Acid	octadecanedioate (C18)_Fatty Acid, Dicarboxylate_Lipid
58	7-HOCA_Sterol_Lipid	dimethylarginine (ADMA + SDMA)_Urea cycle; Arginine and Proline Metabolism_Amino Acid	taurolithocholate 3-sulfate_Secondary Bile Acid Metabolism_Lipid	succinylcarnitine (C4-DC)_TCA Cycle_Energy
59	gamma-glutamylalanine_Gamma-glutamyl Amino Acid_Peptide	N-acetylserine_Glycine, Serine and Threonine Metabolism_Amino Acid	gamma-glutamyl-2-aminobutyrate_Gamma-glutamyl Amino Acid_Peptide	tryptophan betaine_Tryptophan Metabolism_Amino Acid
60	4-allylphenol sulfate_Food Component/Plant_Xenobiotics	5alpha-pregnan-3beta,20alpha-diol disulfate_Progestin Steroids_Lipid	5alpha-pregnan-3beta,20alpha-diol monosulfate (2)_Progestin Steroids_Lipid	androstenediol (3beta,17beta) disulfate (1)_Androgenic Steroids_Lipid
61	androstenediol (3beta,17beta) disulfate (2)_Androgenic Steroids_Lipid	androstenediol (3alpha, 17alpha) monosulfate (3)_Androgenic Steroids_Lipid	N-acetyl-beta-alanine_Pyrimidine Metabolism, Uracil containing_Nucleotide	glycerophosphoethanolamine_Phospholipid Metabolism_Lipid
62	ergothioneine_Food Component/Plant_Xenobiotics	docosapentaenoate (n6 DPA; 22:5n6)_Long Chain Polyunsaturated Fatty Acid (n3 and n6)_Lipid	N-acetylputrescine_Polyamine Metabolism_Amino Acid	palmitoyl sphingomyelin (d18:1/16:0)_Sphingomyelins_Lipid

63	sphingomyelin (d18:1/18:1, d18:2/18:0)_Sphingomyelins_Lipid	13-HODE + 9-HODE_Fatty Acid, Monohydroxy_Lipid	4-cholesten-3-one_Sterol_Lipid	16a-hydroxy DHEA 3-sulfate_Androgenic Steroids_Lipid
64	pregnenolone sulfate_Pregnenolone Steroids_Lipid	cis-4-decenoylcarnitine (C10:1)_Fatty Acid Metabolism (Acyl Carnitine, Monounsaturated)_Lipid	pristanate_Fatty Acid, Branched_Lipid	2,3-dihydroxyisovalerate_Food Component/Plant_Xenobiotics
65	(16 or 17)-methylstearate (a19:0 or i19:0)_Fatty Acid, Branched_Lipid	12,13-DiHOME_Fatty Acid, Dihydroxy_Lipid	9,10-DiHOME_Fatty Acid, Dihydroxy_Lipid	cinnamoylglycine_Food Component/Plant_Xenobiotics
66	(14 or 15)-methylpalmitate (a17:0 or i17:0)_Fatty Acid, Branched_Lipid	1-(1-enyl-palmitoyl)-GPE (P-16:0)*_Lysoplasmalogen_Lipid	1-(1-enyl-stearoyl)-GPE (P-18:0)*_Lysoplasmalogen_Lipid	glycoursodeoxycholate_Secondary Bile Acid Metabolism_Lipid
67	S-methylcysteine_Methionine, Cysteine, SAM and Taurine Metabolism_Amino Acid	glutarate (C5-DC)_Fatty Acid, Dicarboxylate_Lipid	16-hydroxypalmitate_Fatty Acid, Monohydroxy_Lipid	eicosanedioate (C20-DC)_Fatty Acid, Dicarboxylate_Lipid
68	docosadioate (C22-DC)_Fatty Acid, Dicarboxylate_Lipid	carboxyethyl-GABA_Glutamate Metabolism_Amino Acid	isoleucylglycine_Dipeptide_Peptide	4-hydroxy-2-oxoglutaric acid_Fatty Acid, Dicarboxylate_Lipid
69	trimethylamine N-oxide_Phospholipid Metabolism_Lipid	N1-Methyl-2-pyridone-5-carboxamide_Nicotinate and Nicotinamide Metabolism_Cofactors and Vitamins	hydantoin-5-propionate_Histidine Metabolism_Amino Acid	4-hydroxyglutamate_Glutamate Metabolism_Amino Acid
70	butyrate/isobutyrate (4:0)_Short Chain Fatty Acid_Lipid	2-stearoyl-GPE (18:0)*_Lysophospholipid_Lipid	lanthionine_Methionine, Cysteine, SAM and Taurine Metabolism_Amino Acid	N-palmitoylglycine_Fatty Acid Metabolism (Acyl Glycine)_Lipid
71	phosphate_Oxidative Phosphorylation_Energy	S-1-pyrroline-5-carboxylate_Glutamate Metabolism_Amino Acid	2-aminobutyrate_Glutathione Metabolism_Amino Acid	1-stearoyl-GPE (18:0)_Lysophospholipid_Lipid
72	erythronate*_Aminosugar Metabolism_Carbohydrate	1-palmitoyl-2-linoleoyl-GPC (16:0/18:2)_Phosphatidylcholine (PC)_Lipid	1-stearoyl-2-oleoyl-GPE (18:0/18:1)_Phosphatidylethanolamine (PE)_Lipid	1-palmitoyl-2-linoleoyl-GPE (16:0/18:2)_Phosphatidylethanolamine (PE)_Lipid
73	1-stearoyl-2-arachidonoyl-GPC (18:0/20:4)_Phosphatidylcholine (PC)_Lipid	sphingomyelin (d18:2/16:0, d18:1/16:1)*_Sphingomyelins_Lipid	sphingomyelin (d18:1/14:0, d16:1/16:0)*_Sphingomyelins_Lipid	2-hydroxydecanoate_Fatty Acid, Monohydroxy_Lipid
74	glycohyocholate_Secondary Bile Acid Metabolism_Lipid	6-oxopiperidine-2-carboxylate_Lysine Metabolism_Amino Acid	2-aminophenol sulfate_Chemical_Xenobiotics	2-aminooctanoate_Fatty Acid, Amino_Lipid
75	S-methylcysteine sulfoxide_Methionine, Cysteine, SAM and Taurine Metabolism_Amino Acid	2-piperidinone_Food Component/Plant_Xenobiotics	dimethyl sulfone_Chemical_Xenobiotics	N-acetylcarnosine_Histidine Metabolism_Amino Acid
76	formiminoglutamate_Histidine Metabolism_Amino Acid	N-formylanthranilic acid_Tryptophan Metabolism_Amino Acid	5-(galactosylhydroxy)-L-lysine_Lysine Metabolism_Amino Acid	2-aminoheptanoate_Fatty Acid, Amino_Lipid
77	guanidinoacetate_Creatine Metabolism_Amino Acid	bilirubin_Hemoglobin and Porphyrin Metabolism_Cofactors and Vitamins	gamma-glutamylvaline_Gamma-glutamyl Amino Acid_Peptide	glycerol 3-phosphate_Glycerolipid Metabolism_Lipid
78	3-methyl-2-oxobutyrate_Leucine, Isoleucine and Valine Metabolism_Amino Acid	1-(1-enyl-oleoyl)-GPE (P-18:1)*_Lysoplasmalogen_Lipid	isovalerate (C5)_Leucine, Isoleucine and Valine Metabolism_Amino Acid	glutarylcarnitine (C5-DC)_Lysine Metabolism_Amino Acid
79	palmitoylcarnitine (C16)_Fatty Acid Metabolism (Acyl Carnitine, Long Chain Saturated)_Lipid	gamma-glutamylmethionine_Gamma-glutamyl Amino Acid_Peptide	gamma-CEHC_Tocopherol Metabolism_Cofactors and Vitamins	N-palmitoyl-sphingosine (d18:1/16:0)_Ceramides_Lipid
80	methionine sulfone_Methionine, Cysteine, SAM and Taurine Metabolism_Amino Acid	2-methylbutyrylcarnitine (C5)_Leucine, Isoleucine and Valine Metabolism_Amino Acid	O-sulfo-L-tyrosine_Chemical_Xenobiotics	1-linolenoyl-GPC (18:3)*_Lysophospholipid_Lipid

81	1-oleoyl-GPG (18:1)*_Lysophospholipid_Lipid	1-palmitoyl-GPG (16:0)*_Lysophospholipid_Lipid	guaiacol sulfate_Benzoate Metabolism_Xenobiotics	21-hydroxypregnenolone disulfate_Pregnenolone Steroids_Lipid
82	mannitol/sorbitol_Fructose, Mannose and Galactose Metabolism_Carbohydrate	4-methylcatechol sulfate_Benzoate Metabolism_Xenobiotics	3-methyl catechol sulfate (1)_Benzoate Metabolism_Xenobiotics	aconitate [cis or trans]_TCA Cycle_Energy
83	linoleoylcarnitine (C18:2)*_Fatty Acid Metabolism (Acyl Carnitine, Polyunsaturated)_Lipid	pyroglutamine*_Glutamate Metabolism_Amino Acid	X - 21258_NA_NA	X - 15486_NA_NA
84	X - 15461_NA_NA	X - 21286_NA_NA	X - 21310_NA_NA	X - 11441_NA_NA
85	X - 21319_NA_NA	X - 21339_NA_NA	X - 11308_NA_NA	X - 21353_NA_NA
86	X - 21364_NA_NA	X - 13866_NA_NA	X - 21383_NA_NA	X - 21410_NA_NA
87	X - 11444_NA_NA	X - 21441_NA_NA	X - 21448_NA_NA	X - 11850_NA_NA
88	X - 21467_NA_NA	X - 21470_NA_NA	X - 21471_NA_NA	alpha-hydroxyisovalerate_Leucine, Isoleucine and Valine Metabolism_Amino Acid
89	N-acetylglucosamine/N-acetylgalactosamine_Amino sugar Metabolism_Carbohydrate	3-methylglutarylcarnitine (2)_Leucine, Isoleucine and Valine Metabolism_Amino Acid	X - 11299_NA_NA	X - 11372_NA_NA
90	X - 11470_NA_NA	X - 11478_NA_NA	X - 11530_NA_NA	X - 11880_NA_NA
91	X - 12007_NA_NA	X - 12216_NA_NA	X - 12462_NA_NA	X - 12524_NA_NA
92	X - 12729_NA_NA	X - 12798_NA_NA	X - 14056_NA_NA	X - 12844_NA_NA
93	X - 15469_NA_NA	X - 13728_NA_NA	X - 14939_NA_NA	X - 15245_NA_NA
94	X - 16935_NA_NA	X - 16944_NA_NA	X - 16964_NA_NA	X - 18779_NA_NA
95	X - 18913_NA_NA	X - 18922_NA_NA	X - 19141_NA_NA	X - 17653_NA_NA
96	X - 17654_NA_NA	X - 21736_NA_NA	X - 12104_NA_NA	gulonate*_Ascorbate and Aldarate Metabolism_Cofactors and Vitamins
97	sulfate*_Chemical_Xenobiotics	X - 17676_NA_NA	X - 15503_NA_NA	X - 12193_NA_NA
98	X - 21829_NA_NA	N-methylpipecolate_Bacterial/Fungal_Xenobiotics	ferulic acid 4-sulfate_Food Component/Plant_Xenobiotics	2-palmitoleoyl-GPC* (16:1)*_Lysophospholipid_Lipid
99	9-hydroxystearate_Fatty Acid, Monohydroxy_Lipid	sphingomyelin (d18:1/24:1, d18:2/24:0)*_Sphingomyelins_Lipid	sphingomyelin (d18:2/14:0, d18:1/14:1)*_Sphingomyelins_Lipid	X - 18887_NA_NA
100	X - 22162_NA_NA	X - 12680_NA_NA	X - 12101_NA_NA	X - 18899_NA_NA
101	X - 18921_NA_NA	X - 12100_NA_NA	X - 12112_NA_NA	X - 13431_NA_NA
102	X - 16397_NA_NA	X - 16580_NA_NA	bilirubin (E,Z or Z,E)*_Hemoglobin and Porphyrin Metabolism_Cofactors and Vitamins	X - 12026_NA_NA
103	X - 12221_NA_NA	X - 12906_NA_NA	X - 13553_NA_NA	X - 17351_NA_NA
104	X - 17357_NA_NA	X - 22771_NA_NA	mannose_Fructose, Mannose and Galactose Metabolism_Carbohydrate	myristoleoylcarnitine (C14:1)*_Fatty Acid Metabolism (Acyl Carnitine, Monounsaturated)_Lipid
105	N-acetyltaurine_Methionine, Cysteine, SAM and Taurine Metabolism_Amino Acid	fructose_Fructose, Mannose and Galactose Metabolism_Carbohydrate	arabonate/xylonate_Pentose Metabolism_Carbohydrate	1-oleoyl-GPC (18:1)_Lysophospholipid_Lipid
106	1-dihomo-linolenylglycerol (20:3)_Monoacylglycerol_Lipid	N1-methylinosine_Purine Metabolism, (Hypo)Xanthine/Inosine containing_Nucleotide	dopamine 3-O-sulfate_Tyrosine Metabolism_Amino Acid	tyramine O-sulfate_Tyrosine Metabolism_Amino Acid
107	pyrraline_Food Component/Plant_Xenobiotics	4-hydroxychlorothalonil_Chemical_Xenobiotics	4-vinylguaiacol sulfate_Food Component/Plant_Xenobiotics	3-hydroxypyridine sulfate_Chemical_Xenobiotics

108	4-methoxyphenol sulfate_Tyrosine Metabolism_Amino Acid	sphingomyelin (d18:1/20:0, d16:1/22:0)*_Sphingomyelins_Lipid	sphingomyelin (d18:1/20:1, d18:2/20:0)*_Sphingomyelins_Lipid	behenoyl sphingomyelin (d18:1/22:0)*_Sphingomyelins_Lipid
109	sphingomyelin (d18:1/22:1, d18:2/22:0, d16:1/24:1)*_Sphingomyelins_Lipid	3-methoxycatechol sulfate (1)_Benzoate Metabolism_Xenobiotics	C-glycosyltryptophan_Tryptophan Metabolism_Amino Acid	glycerophosphoglycerol_Glycerolipid Metabolism_Lipid
110	arabitol/xylitol_Pentose Metabolism_Carbohydrate	X - 23593_NA_NA	X - 23636_NA_NA	X - 23639_NA_NA
111	X - 23665_NA_NA	X - 23666_NA_NA	X - 23680_NA_NA	X - 11315_NA_NA
112	1-lignoceroyl-GPC (24:0)_Lysophospholipid_Lipid	X - 23739_NA_NA	alpha-ketobutyrate_Methionine, Cysteine, SAM and Taurine Metabolism_Amino Acid	X - 23782_NA_NA
113	dimethylglycine_Glycine, Serine and Threonine Metabolism_Amino Acid	asparagine_Alanine and Aspartate Metabolism_Amino Acid	creatinine_Creatine Metabolism_Amino Acid	X - 23997_NA_NA
114	glycosyl-N-stearoyl-sphingosine (d18:1/18:0)_Hexosylceramides (HCER)_Lipid	2-hydroxybutyrate/2-hydroxyisobutyrate_Glutathione Metabolism_Amino Acid	X - 24243_NA_NA	1-palmitoleoylglycerol (16:1)*_Monoacylglycerol_Lipid
115	sphingomyelin (d17:1/16:0, d18:1/15:0, d16:1/17:0)*_Sphingomyelins_Lipid	palmitoyl dihydrosphingomyelin (d18:0/16:0)*_Dihydrosphingomyelins_Lipid	sphingomyelin (d18:2/23:0, d18:1/23:1, d17:1/24:1)*_Sphingomyelins_Lipid	tricosanoyl sphingomyelin (d18:1/23:0)*_Sphingomyelins_Lipid
116	sphingomyelin (d18:2/24:1, d18:1/24:2)*_Sphingomyelins_Lipid	1-stearoyl-2-oleoyl-GPC (18:0/18:1)_Phosphatidylcholine (PC)_Lipid	1-stearoyl-2-arachidonoyl-GPE (18:0/20:4)_Phosphatidylethanolamine (PE)_Lipid	1-stearoyl-2-arachidonoyl-GPI (18:0/20:4)_Phosphatidylinositol (PI)_Lipid
117	1-palmitoyl-2-linoleoyl-GPI (16:0/18:2)_Phosphatidylinositol (PI)_Lipid	1-stearoyl-2-linoleoyl-GPC (18:0/18:2)*_Phosphatidylcholine (PC)_Lipid	1-palmitoyl-2-oleoyl-GPC (16:0/18:1)_Phosphatidylcholine (PC)_Lipid	1-palmitoyl-2-arachidonoyl-GPC (16:0/20:4n6)_Phosphatidylcholine (PC)_Lipid
118	1-palmitoyl-2-arachidonoyl-GPE (16:0/20:4)*_Phosphatidylethanolamine (PE)_Lipid	1-palmitoyl-2-docosahexaenoyl-GPE (16:0/22:6)*_Phosphatidylethanolamine (PE)_Lipid	1-stearoyl-2-docosahexaenoyl-GPE (18:0/22:6)*_Phosphatidylethanolamine (PE)_Lipid	1-palmitoyl-2-arachidonoyl-GPI (16:0/20:4)*_Phosphatidylinositol (PI)_Lipid
119	1-stearoyl-2-linoleoyl-GPI (18:0/18:2)_Phosphatidylinositol (PI)_Lipid	1-palmitoyl-2-palmitoleoyl-GPC (16:0/16:1)*_Phosphatidylcholine (PC)_Lipid	gamma-tocopherol/beta-tocopherol_Tocopherol Metabolism_Cofactors and Vitamins	1-(1-enyl-palmitoyl)-GPC (P-16:0)*_Lysoplasmalogen_Lipid
120	1-(1-enyl-stearoyl)-2-arachidonoyl-GPE (P-18:0/20:4)*_Plasmalogen_Lipid	1-(1-enyl-palmitoyl)-2-oleoyl-GPE (P-16:0/18:1)*_Plasmalogen_Lipid	1-(1-enyl-palmitoyl)-2-oleoyl-GPC (P-16:0/18:1)*_Plasmalogen_Lipid	X - 24295_NA_NA
121	sphingomyelin (d18:1/21:0, d17:1/22:0, d16:1/23:0)*_Sphingomyelins_Lipid	X - 24307_NA_NA	X - 24337_NA_NA	1,2-dilinoleoyl-GPC (18:2/18:2)_Phosphatidylcholine (PC)_Lipid
122	N-palmitoyl-sphinganine (d18:0/16:0)_Dihydroceramides_Lipid	sphinganine-1-phosphate_Sphingolipid Synthesis_Lipid	1-palmitoyl-2-docosahexaenoyl-GPC (16:0/22:6)_Phosphatidylcholine (PC)_Lipid	1-stearoyl-2-docosahexaenoyl-GPC (18:0/22:6)_Phosphatidylcholine (PC)_Lipid
123	1-(1-enyl-stearoyl)-2-oleoyl-GPE (P-18:0/18:1)_Plasmalogen_Lipid	sphingomyelin (d18:1/17:0, d17:1/18:0, d19:1/16:0)_Sphingomyelins_Lipid	1-palmitoyl-2-stearoyl-GPC (16:0/18:0)_Phosphatidylcholine (PC)_Lipid	X - 24431_NA_NA
124	X - 24432_NA_NA	1-palmitoyl-2-oleoyl-GPI (16:0/18:1)*_Phosphatidylinositol (PI)_Lipid	1-(1-enyl-palmitoyl)-2-arachidonoyl-GPE (P-16:0/20:4)*_Plasmalogen_Lipid	1-(1-enyl-palmitoyl)-2-linoleoyl-GPE (P-16:0/18:2)*_Plasmalogen_Lipid
125	1-(1-enyl-palmitoyl)-2-linoleoyl-GPC (P-	1-oleoyl-2-linoleoyl-GPE (18:1/18:2)*_Phosphatidylethanolamine (PE)_Lipid	1-(1-enyl-palmitoyl)-2-arachidonoyl-GPC (P-	1-linoleoyl-GPA (18:2)*_Lysophospholipid_Lipid

	16:0/18:2)*_Plasmalogen_Lipid		16:0/20:4)*_Plasmalogen_Lipid	
126	1-oleoyl-2-docosahexaenoyl-GPC (18:1/22:6)*_Phosphatidylcholine (PC)_Lipid	lactate_Glycolysis, Gluconeogenesis, and Pyruvate Metabolism_Carbohydrate	1-linoleoyl-2-arachidonoyl-GPC (18:2/20:4n6)*_Phosphatidylcholine (PC)_Lipid	1-(1-enyl-palmitoyl)-2-palmitoleoyl-GPC (P-16:0/16:1)*_Plasmalogen_Lipid
127	1-(1-enyl-palmitoyl)-2-palmitoyl-GPC (P-16:0/16:0)*_Plasmalogen_Lipid	1-stearoyl-2-oleoyl-GPI (18:0/18:1)*_Phosphatidylinositol (PI)_Lipid	1-(1-enyl-stearoyl)-2-linoleoyl-GPE (P-18:0/18:2)*_Plasmalogen_Lipid	X - 24455_NA_NA
128	X - 24473_NA_NA	alpha-ketoglutarate_TCA Cycle_Energy	X - 24544_NA_NA	X - 24545_NA_NA
129	X - 24556_NA_NA	X - 24588_NA_NA	2-hydroxylaurate_Fatty Acid, Monohydroxy_Lipid	palmitoylcholine_Fatty Acid Metabolism (Acyl Choline)_Lipid
130	glycochenodeoxycholate 3-sulfate_Primary Bile Acid Metabolism_Lipid	glycodeoxycholate 3-sulfate_Secondary Bile Acid Metabolism_Lipid	glycochenodeoxycholate glucuronide (1)_Primary Bile Acid Metabolism_Lipid	(S)-3-hydroxybutyrylcarnitine_Fatty Acid Metabolism (Acyl Carnitine, Hydroxy)_Lipid
131	N1,N12-diacetylspermine_Polyamine Metabolism_Amino Acid	adipoylcarnitine (C6-DC)_Fatty Acid Metabolism (Acyl Carnitine, Dicarboxylate)_Lipid	glutamine_Glutamate Metabolism_Amino Acid	lactosyl-N-palmitoyl-sphingosine (d18:1/16:0)_Lactosylceramides (LCER)_Lipid
132	glycosyl-N-palmitoyl-sphingosine (d18:1/16:0)_Hexosylceramides (HCER)_Lipid	3-hydroxy-3-methylglutarate_Mevalonate Metabolism_Lipid	X - 24728_NA_NA	1-linoleoyl-2-linolenoyl-GPC (18:2/18:3)*_Phosphatidylcholine (PC)_Lipid
133	1-palmitoleoyl-2-linolenoyl-GPC (16:1/18:3)*_Phosphatidylcholine (PC)_Lipid	1-myristoyl-2-arachidonoyl-GPC (14:0/20:4)*_Phosphatidylcholine (PC)_Lipid	palmitoleoylcarnitine (C16:1)*_Fatty Acid Metabolism (Acyl Carnitine, Monounsaturated)_Lipid	pimeloylcarnitine/3-methyladipoylcarnitine (C7-DC)_Fatty Acid Metabolism (Acyl Carnitine, Dicarboxylate)_Lipid
134	3-hydroxyhexanoate_Fatty Acid, Monohydroxy_Lipid	thioproline_Chemical_Xenobiotics	caffeic acid sulfate_Food Component/Plant_Xenobiotics	tryptophan_Tryptophan Metabolism_Amino Acid
135	3-hydroxybutyrate (BHBA)_Ketone Bodies_Lipid	3beta-hydroxy-5-cholestenoate_Sterol_Lipid	1-linoleoyl-GPG (18:2)*_Lysophospholipid_Lipid	vanillactate_Tyrosine Metabolism_Amino Acid
136	diacylglycerol (16:1/18:2 [2], 16:0/18:3 [1])*_Diacylglycerol_Lipid	palmitoleoyl-linoleoyl-glycerol (16:1/18:2) [1]*_Diacylglycerol_Lipid	N-stearoyl-sphingosine (d18:1/18:0)*_Ceramides_Lipid	beta-alanine_Pyrimidine Metabolism, Uracil containing_Nucleotide
137	gamma-glutamyl-alpha-lysine_Gamma-glutamyl Amino Acid_Peptide	2-oxoarginine*_Urea cycle; Arginine and Proline Metabolism_Amino Acid	phenylpyruvate_Phenylalanine Metabolism_Amino Acid	glutamate_Glutamate Metabolism_Amino Acid
138	lignoceroyl sphingomyelin (d18:1/24:0)_Sphingomyelins_Lipid	behenoyl dihydrosphingomyelin (d18:0/22:0)*_Dihydrosphingomyelins_Lipid	myristoyl dihydrosphingomyelin (d18:0/14:0)*_Dihydrosphingomyelins_Lipid	lactosyl-N-nervonoyl-sphingosine (d18:1/24:1)*_Lactosylceramides (LCER)_Lipid
139	N-behenoyl-sphingadienine (d18:2/22:0)*_Ceramides_Lipid	palmitoyl-docosahexaenoyl-glycerol (16:0/22:6) [1]*_Diacylglycerol_Lipid	N-palmitoyl-sphingadienine (d18:2/16:0)*_Ceramides_Lipid	lactosyl-N-behenoyl-sphingosine (d18:1/22:0)*_Lactosylceramides (LCER)_Lipid
140	ceramide (d18:1/14:0, d16:1/16:0)*_Ceramides_Lipid	ceramide (d16:1/24:1, d18:1/22:1)*_Ceramides_Lipid	ceramide (d18:1/20:0, d16:1/22:0, d20:1/18:0)*_Ceramides_Lipid	ceramide (d18:2/24:1, d18:1/24:2)*_Ceramides_Lipid
141	glycosyl-N-(2-hydroxynervonoyl)-sphingosine (d18:1/24:1(2OH))*_Hexosylceramides (HCER)_Lipid	glycosyl ceramide (d18:2/24:1, d18:1/24:2)*_Hexosylceramides (HCER)_Lipid	argininate*_Urea cycle; Arginine and Proline Metabolism_Amino Acid	sphingomyelin (d18:0/18:0, d19:0/17:0)*_Dihydrosphingomyelins_Lipid
142	sphingomyelin (d18:2/18:1)*_Sphingomyelins_Lipid	sphingomyelin (d18:1/19:0, d19:1/18:0)*_Sphingomyelins_Lipid	sphingomyelin (d18:1/22:2, d18:2/22:1,	sphingomyelin (d18:1/25:0, d19:0/24:1, d20:1/23:0,

			d16:1/24:2)*_Sphingomyelins_Lipid	d19:1/24:0)*_Sphingomyelins_Lipid
143	sphingomyelin (d18:2/24:2)*_Sphingomyelins_Lipid	sphingomyelin (d18:2/21:0, d16:2/23:0)*_Sphingomyelins_Lipid	sphingomyelin (d18:1/20:2, d18:2/20:1, d16:1/22:2)*_Sphingomyelins_Lipid	sphingomyelin (d17:2/16:0, d18:2/15:0)*_Sphingomyelins_Lipid
144	arabinose_Pentose Metabolism_Carbohydrate	margaroylcarnitine (C17)*_Fatty Acid Metabolism (Acyl Carnitine, Long Chain Saturated)_Lipid	arachidoylcarnitine (C20)*_Fatty Acid Metabolism (Acyl Carnitine, Long Chain Saturated)_Lipid	behenoylcarnitine (C22)*_Fatty Acid Metabolism (Acyl Carnitine, Long Chain Saturated)_Lipid
145	lignoceroylcarnitine (C24)*_Fatty Acid Metabolism (Acyl Carnitine, Long Chain Saturated)_Lipid	cerotoylcarnitine (C26)*_Fatty Acid Metabolism (Acyl Carnitine, Long Chain Saturated)_Lipid	ximenoylcarnitine (C26:1)*_Fatty Acid Metabolism (Acyl Carnitine, Monounsaturated)_Lipid	arachidonoylcarnitine (C20:4)_Fatty Acid Metabolism (Acyl Carnitine, Polyunsaturated)_Lipid
146	eicosenoylcarnitine (C20:1)*_Fatty Acid Metabolism (Acyl Carnitine, Monounsaturated)_Lipid	dihomo-linoleoylcarnitine (C20:2)*_Fatty Acid Metabolism (Acyl Carnitine, Polyunsaturated)_Lipid	dihomo-linolenoylcarnitine (C20:3n3 or 6)*_Fatty Acid Metabolism (Acyl Carnitine, Polyunsaturated)_Lipid	nervonoylcarnitine (C24:1)*_Fatty Acid Metabolism (Acyl Carnitine, Monounsaturated)_Lipid
147	2'-O-methylcytidine_Pyrimidine Metabolism, Cytidine containing_Nucleotide	perfluorooctanesulfonate (PFOS)_Chemical_Xenobiotics	beta-cryptoxanthin_Vitamin A Metabolism_Cofactors and Vitamins	carotene diol (1)_Vitamin A Metabolism_Cofactors and Vitamins
148	carotene diol (2)_Vitamin A Metabolism_Cofactors and Vitamins	carotene diol (3)_Vitamin A Metabolism_Cofactors and Vitamins	hexadecadienoate (16:2n6)_Long Chain Polyunsaturated Fatty Acid (n3 and n6)_Lipid	N-palmitoylserine_Endocannabinoid_Lipid
149	2-hydroxybehenate_Fatty Acid, Monohydroxy_Lipid	N-oleoylserine_Endocannabinoid_Lipid	N,N,N-trimethyl-5-aminovalerate_Lysine Metabolism_Amino Acid	X - 24947_NA_NA
150	X - 24951_NA_NA	3-methylglutaconate_Leucine, Isoleucine and Valine Metabolism_Amino Acid	X - 24970_NA_NA	nisinate (24:6n3)_Long Chain Polyunsaturated Fatty Acid (n3 and n6)_Lipid
151	(N(1) + N(8))-acetylspermidine_Polyamine Metabolism_Amino Acid	glycine_Glycine, Serine and Threonine Metabolism_Amino Acid	gluconate_Food Component/Plant_Xenobiotics	histidine_Histidine Metabolism_Amino Acid
152	hypotaurine_Methionine, Cysteine, SAM and Taurine Metabolism_Amino Acid	phytanate_Food Component/Plant_Xenobiotics	leucine_Leucine, Isoleucine and Valine Metabolism_Amino Acid	dihydroorotate_Pyrimidine Metabolism, Orotate containing_Nucleotide
153	uridine_Pyrimidine Metabolism, Uracil containing_Nucleotide	trans-urocanate_Histidine Metabolism_Amino Acid	2-aminoadipate_Lysine Metabolism_Amino Acid	2-hydroxynervonate*_Fatty Acid, Monohydroxy_Lipid
154	2-hydroxyarachidate*_Fatty Acid, Monohydroxy_Lipid	5,6-dihydrouridine_Pyrimidine Metabolism, Uracil containing_Nucleotide	ribulonate/xylulonate/lyxonate*_Pentose Metabolism_Carbohydrate	octadecadienedioate (C18:2-DC)*_Fatty Acid, Dicarboxylate_Lipid
155	octadecenedioate (C18:1-DC)_Fatty Acid, Dicarboxylate_Lipid	hexadecenedioate (C16:1-DC)*_Fatty Acid, Dicarboxylate_Lipid	octadecenedioylcarnitine (C18:1-DC)*_Fatty Acid Metabolism (Acyl Carnitine, Dicarboxylate)_Lipid	heptenedioate (C7:1-DC)*_Fatty Acid, Dicarboxylate_Lipid
156	octadecanedioylcarnitine (C18-DC)*_Fatty Acid Metabolism (Acyl Carnitine, Dicarboxylate)_Lipid	1-ribosyl-imidazoleacetate*_Histidine Metabolism_Amino Acid	3-carboxy-4-methyl-5-pentyl-2-furanpropionate (3-CMPFP)_Fatty Acid, Dicarboxylate_Lipid	glucuronide of C12H22O4 (1)*_Partially Characterized Molecules_Partially Characterized Molecules
157	N-acetyl-2-aminooctanoate*_Fatty Acid, Amino_Lipid	hydroxyasparagine_Alanine and Aspartate Metabolism_Amino Acid	3-hydroxystachydrine*_Food Component/Plant_Xenobiotics	glyco-beta-muricholate_Primary Bile Acid Metabolism_Lipid
158	perfluorooctanoate (PFOA)_Chemical_Xenobiotics	alpha-ketoglutaramate*_Glutamate Metabolism_Amino Acid	cysteinylglycine disulfide*_Glutathione Metabolism_Amino Acid	gamma-glutamylcitrulline*_Gamma-glutamyl Amino Acid_Peptide
159	glycine conjugate of C10H14O2 (1)*_Partially Characterized Molecules_Partially Characterized Molecules	X - 25172_NA_NA	sphingomyelin (d17:1/14:0, d16:1/15:0)*_Sphingomyelins_Lipid	dodecadienoate (12:2)*_Fatty Acid, Dicarboxylate_Lipid

160	hydroxy-CMPF*_Fatty Acid, Dicarboxylate_Lipid	indoleacetoylcarnitine*_Tryptophan Metabolism_Amino Acid	N-acetyl-isoputreanine_Polyamine Metabolism_Amino Acid	1-carboxyethylvaline_Leucine, Isoleucine and Valine Metabolism_Amino Acid
161	1-carboxyethylphenylalanine_Phenylalanine Metabolism_Amino Acid	2,3-dihydroxy-5-methylthio-4-pentenoate (DMTPA)*_Methionine, Cysteine, SAM and Taurine Metabolism_Amino Acid	tetradecadienoate (14:2)*_Long Chain Polyunsaturated Fatty Acid (n3 and n6)_Lipid	hydroxypalmitoyl sphingomyelin (d18:1/16:0(OH))_Sphingomyelins_Lipid
162	3-amino-2-piperidone_Urea cycle; Arginine and Proline Metabolism_Amino Acid	N6-methyllysine_Lysine Metabolism_Amino Acid	N6,N6-dimethyllysine_Lysine Metabolism_Amino Acid	cis-4-decenoate (10:1n6)*_Medium Chain Fatty Acid_Lipid
163	taurochenodeoxycholic acid 3-sulfate_Secondary Bile Acid Metabolism_Lipid	tridecenedioate (C13:1-DC)*_Fatty Acid, Dicarboxylate_Lipid	pregnenetriol disulfate*_Pregnenolone Steroids_Lipid	pregnenetriol sulfate*_Pregnenolone Steroids_Lipid
164	eicosenedioate (C20:1-DC)*_Fatty Acid, Dicarboxylate_Lipid	1-methyl-5-imidazoleacetate_Histidine Metabolism_Amino Acid	hydroxy-N6,N6,N6-trimethyllysine*_Lysine Metabolism_Amino Acid	palmitoyl-sphingosine-phosphoethanolamine (d18:1/16:0)_Ceramide PEs_Lipid
165	cholesterol_Sterol_Lipid	2,6-dihydroxybenzoic acid_Drug - Topical Agents_Xenobiotics	picolinoylglycine_Fatty Acid Metabolism (Acyl Glycine)_Lipid	N,N-dimethylalanine_Alanine and Aspartate Metabolism_Amino Acid
166	ethyl alpha-glucopyranoside_Food Component/Plant_Xenobiotics	metabolonic lactone sulfate_Partially Characterized Molecules_Partially Characterized Molecules	branched chain 14:0 dicarboxylic acid_Fatty Acid, Dicarboxylate_Lipid	(2 or 3)-decenoate (10:1n7 or n8)_Medium Chain Fatty Acid_Lipid
167	3-indoleglyoxylic acid_Food Component/Plant_Xenobiotics	deoxycholic acid glucuronide_Secondary Bile Acid Metabolism_Lipid	undecenoylcarnitine (C11:1)_Fatty Acid Metabolism (Acyl Carnitine, Monounsaturated)_Lipid	branched-chain, straight-chain, or cyclopropyl 10:1 fatty acid (1)*_Partially Characterized Molecules_Partially Characterized Molecules
168	branched-chain, straight-chain, or cyclopropyl 10:1 fatty acid (2)*_Partially Characterized Molecules_Partially Characterized Molecules	glutamine conjugate of C6H10O2 (1)*_Partially Characterized Molecules_Partially Characterized Molecules	branched-chain, straight-chain, or cyclopropyl 12:1 fatty acid*_Partially Characterized Molecules_Partially Characterized Molecules	vanillic acid glycine_Food Component/Plant_Xenobiotics
169	4-chlorobenzoic acid_Chemical_Xenobiotics	2-O-methylascorbic acid_Ascorbate and Aldarate Metabolism_Cofactors and Vitamins	phenylalanine_Phenylalanine Metabolism_Amino Acid	linoleate (18:2n6)_Long Chain Polyunsaturated Fatty Acid (n3 and n6)_Lipid

Table S2. The list of 205 metabolites out of the 884, which didn't enter the models.

	Column1	Column2	Column3	Column4
0	inosine_Purine Metabolism, (Hypo)Xanthine/Inosine containing_Nucleotide	4-allylcatechol sulfate_Benzoate Metabolism_Xenobiotics	X - 16124_NA_NA	X - 25217_NA_NA
1	X - 21752_NA_NA	hexanoylglutamine_Fatty Acid Metabolism (Acyl Glutamine)_Lipid	cysteine-glutathione disulfide_Glutathione Metabolism_Amino Acid	5alpha-pregnan-diol disulfate_Progestin Steroids_Lipid
2	3-hydroxyhippurate_Benzoate Metabolism_Xenobiotics	4-acetamidophenylglucuronide_Drug - Analgesics, Anesthetics_Xenobiotics	androsterone glucuronide_Androgenic Steroids_Lipid	xylose_Pentose Metabolism_Carbohydrate
3	X - 11843_NA_NA	2-acetamidophenol sulfate_Drug - Analgesics, Anesthetics_Xenobiotics	N,N,N-trimethyl-alanylproline betaine (TMAP)_Urea cycle; Arginine and Proline Metabolism_Amino Acid	X - 24546_NA_NA
4	androstenediol (3alpha, 17alpha) monosulfate (2)_Androgenic Steroids_Lipid	linoleoylcholine*_Fatty Acid Metabolism (Acyl Choline)_Lipid	docosahexaenoylcholine_Fatty Acid Metabolism (Acyl Choline)_Lipid	X - 12544_NA_NA
5	solanidine_Food Component/Plant_Xenobiotics	acesulfame_Food Component/Plant_Xenobiotics	cytosine_Pyrimidine Metabolism, Cytidine containing_Nucleotide	nonanoylcarnitine (C9)_Fatty Acid Metabolism (Acyl Carnitine, Medium Chain)_Lipid
6	X - 17185_NA_NA	X - 12812_NA_NA	1,2-dilinoleoyl-GPE (18:2/18:2)*_Phosphatidylethanolamine (PE)_Lipid	X - 21796_NA_NA
7	X - 13684_NA_NA	palmitate (16:0)_Long Chain Saturated Fatty Acid_Lipid	1,3,7-trimethylurate_Xanthine Metabolism_Xenobiotics	sphingadienine_Sphingolipid Synthesis_Lipid
8	X - 15728_NA_NA	gentisate_Tyrosine Metabolism_Amino Acid	4-ethylcatechol sulfate_Benzoate Metabolism_Xenobiotics	2-ketocaprylate_Leucine, Isoleucine and Valine Metabolism_Amino Acid
9	X - 12849_NA_NA	glucuronide of C10H18O2 (7)*_Partially Characterized Molecules_Partially Characterized Molecules	linolenate (18:3n3 or 3n6)_Long Chain Polyunsaturated Fatty Acid (n3 and n6)_Lipid	X - 21834_NA_NA
10	3-methoxycatechol sulfate (2)_Benzoate Metabolism_Xenobiotics	orotate_Pyrimidine Metabolism, Orotate containing_Nucleotide	X - 17612_NA_NA	10-heptadecenoate (17:1n7)_Long Chain Monounsaturated Fatty Acid_Lipid
11	X - 21742_NA_NA	X - 24849_NA_NA	margarate (17:0)_Long Chain Saturated Fatty Acid_Lipid	octanoylcarnitine (C8)_Fatty Acid Metabolism (Acyl Carnitine, Medium Chain)_Lipid
12	X - 17343_NA_NA	X - 24571_NA_NA	suberate (C8-DC)_Fatty Acid, Dicarboxylate_Lipid	stearoylcholine*_Fatty Acid Metabolism (Acyl Choline)_Lipid
13	nicotinamide_Nicotinate and Nicotinamide Metabolism_Cofactors and Vitamins	glycoursodeoxycholic acid sulfate (1)_Secondary Bile Acid Metabolism_Lipid	syringol sulfate_Food Component/Plant_Xenobiotics	glucuronide of piperine metabolite C17H21NO3 (4)*_Food Component/Plant_Xenobiotics
14	X - 24699_NA_NA	tauroursodeoxycholate_Secondary Bile Acid Metabolism_Lipid	eugenol sulfate_Food Component/Plant_Xenobiotics	lithocholate sulfate (1)_Secondary Bile Acid Metabolism_Lipid
15	1-stearoyl-2-linoleoyl-GPE (18:0/18:2)*_Phosphatidylethanolamine (PE)_Lipid	5-acetylamino-6-amino-3-methyluracil_Xanthine Metabolism_Xenobiotics	phenylacetylcarnitine_Acetylated Peptides_Peptide	umbelliferone sulfate_Food Component/Plant_Xenobiotics
16	X - 24549_NA_NA	3-methylxanthine_Xanthine Metabolism_Xenobiotics	oleate/vaccenate (18:1)_Long Chain Monounsaturated Fatty Acid_Lipid	3-(3-hydroxyphenyl)propionate_Benzoate Metabolism_Xenobiotics

17	taurohyocholate*_Secondary Bile Acid Metabolism_Lipid	imidazole propionate_Histidine Metabolism_Amino Acid	N-acetylleucine_Leucine, Isoleucine and Valine Metabolism_Amino Acid	X - 21607_NA_NA
18	6-hydroxyindole sulfate_Chemical_Xenobiotics	o-cresol sulfate_Benzoate Metabolism_Xenobiotics	deoxycholate_Secondary Bile Acid Metabolism_Lipid	sebacate (C10-DC)_Fatty Acid, Dicarboxylate_Lipid
19	hydroquinone sulfate_Drug - Topical Agents_Xenobiotics	X - 21803_NA_NA	X - 11442_NA_NA	dihydroferulate_Food Component/Plant_Xenobiotics
20	erythritol_Food Component/Plant_Xenobiotics	linolenoylcarnitine (C18:3)*_Fatty Acid Metabolism (Acyl Carnitine, Polyunsaturated)_Lipid	1,7-dimethylurate_Xanthine Metabolism_Xenobiotics	suberoylcarnitine (C8-DC)_Fatty Acid Metabolism (Acyl Carnitine, Dicarboxylate)_Lipid
21	cysteinylglycine_Glutathione Metabolism_Amino Acid	5alpha-pregnan-3beta,20beta-diol monosulfate (1)_Progestin Steroids_Lipid	maltose_Glycogen Metabolism_Carbohydrate	X - 11858_NA_NA
22	1-methylxanthine_Xanthine Metabolism_Xenobiotics	3,4-methyleneheptanoate_Food Component/Plant_Xenobiotics	ADSGEGDFXAEGGGVR*_Fibrinogen Cleavage Peptide_Peptide	caffeine_Xanthine Metabolism_Xenobiotics
23	sphinganine_Sphingolipid Synthesis_Lipid	ursodeoxycholate_Secondary Bile Acid Metabolism_Lipid	X - 12411_NA_NA	N-acetyl-1-methylhistidine*_Histidine Metabolism_Amino Acid
24	5alpha-androstan-3beta,17alpha-diol disulfate_Androgenic Steroids_Lipid	2-hydroxypalmitate_Fatty Acid, Monohydroxy_Lipid	X - 23641_NA_NA	chiro-inositol_Inositol Metabolism_Lipid
25	epiandrosterone sulfate_Androgenic Steroids_Lipid	X - 21442_NA_NA	cholate_Primary Bile Acid Metabolism_Lipid	N-ethylglycinexylidide_Drug - Analgesics, Anesthetics_Xenobiotics
26	serotonin_Tryptophan Metabolism_Amino Acid	alliin_Food Component/Plant_Xenobiotics	glycodeoxycholate_Secondary Bile Acid Metabolism_Lipid	X - 11522_NA_NA
27	aspartate_Alanine and Aspartate Metabolism_Amino Acid	AMP_Purine Metabolism, Adenine containing_Nucleotide	dihomo-linolenoyl-choline_Fatty Acid Metabolism (Acyl Choline)_Lipid	X - 11849_NA_NA
28	2-hydroxyhippurate (salicylurate)_Benzoate Metabolism_Xenobiotics	methyl glucopyranoside (alpha + beta)_Food Component/Plant_Xenobiotics	2-hydroxyacetaminophen sulfate*_Drug - Analgesics, Anesthetics_Xenobiotics	docosahexaenoylcarnitine (C22:6)*_Fatty Acid Metabolism (Acyl Carnitine, Polyunsaturated)_Lipid
29	p-cresol glucuronide*_Tyrosine Metabolism_Amino Acid	paraxanthine_Xanthine Metabolism_Xenobiotics	N-methylproline_Urea cycle; Arginine and Proline Metabolism_Amino Acid	X - 18901_NA_NA
30	1,2,3-benzenetriol sulfate (2)_Chemical_Xenobiotics	4-acetamidophenol_Drug - Analgesics, Anesthetics_Xenobiotics	glucuronide of C10H18O2 (8)*_Partially Characterized Molecules_Partially Characterized Molecules	(S)-a-amino-omega-caprolactam_Food Component/Plant_Xenobiotics
31	2-methoxyacetaminophen sulfate*_Drug - Analgesics, Anesthetics_Xenobiotics	N6-methyladenosine_Purine Metabolism, Adenine containing_Nucleotide	4-hydroxycoumarin_Drug - Cardiovascular_Xenobiotics	X - 24952_NA_NA
32	X - 12407_NA_NA	N-acetylvaline_Leucine, Isoleucine and Valine Metabolism_Amino Acid	caprylate (8:0)_Medium Chain Fatty Acid_Lipid	X - 12117_NA_NA
33	docosahexaenoate (DHA; 22:6n3)_Long Chain Polyunsaturated Fatty Acid (n3 and n6)_Lipid	4-methylguaiacol sulfate_Benzoate Metabolism_Xenobiotics	X - 12740_NA_NA	vanillic alcohol sulfate_Tyrosine Metabolism_Amino Acid
34	X - 17690_NA_NA	2-methoxyacetaminophen glucuronide*_Drug - Analgesics, Anesthetics_Xenobiotics	N-acetylmethionine_Methionine, Cysteine, SAM and Taurine Metabolism_Amino Acid	X - 24972_NA_NA

35	salicylate_Drug - Topical Agents_Xenobiotics	X - 17348_NA_NA	X - 21821_NA_NA	isoursodeoxycholate_Secondary Bile Acid Metabolism_Lipid
36	X - 17655_NA_NA	X - 24565_NA_NA	sphingomyelin (d18:0/20:0, d16:0/22:0)*_Dihydrosphingomyelins_Lipid	X - 07765_NA_NA
37	orotidine_Pyrimidine Metabolism, Orotate containing_Nucleotide	3-(methylthio)acetaminophen sulfate*_Drug - Analgesics, Anesthetics_Xenobiotics	1-methylhistidine_Histidine Metabolism_Amino Acid	sphingomyelin (d18:2/23:1)*_Sphingomyelins_Lipid
38	3-phosphoglycerate_Glycolysis, Gluconeogenesis, and Pyruvate Metabolism_Carbohydrate	beta-citrylglutamate_Glutamate Metabolism_Amino Acid	X - 21815_NA_NA	N-delta-acetylornithine_Urea cycle; Arginine and Proline Metabolism_Amino Acid
39	sulfate of piperine metabolite C16H19NO3 (2)*_Food Component/Plant_Xenobiotics	sulfate of piperine metabolite C16H19NO3 (3)*_Food Component/Plant_Xenobiotics	DSGEGDFXAEGGGVR*_Fibrinogen Cleavage Peptide_Peptide	4-acetaminophen sulfate_Drug - Analgesics, Anesthetics_Xenobiotics
40	X - 12818_NA_NA	cystine_Methionine, Cysteine, SAM and Taurine Metabolism_Amino Acid	X - 11483_NA_NA	X - 12730_NA_NA
41	maltol sulfate_Food Component/Plant_Xenobiotics	X - 15674_NA_NA	X - 11852_NA_NA	saccharin_Food Component/Plant_Xenobiotics
42	X - 12847_NA_NA	2-hydroxyoctanoate_Fatty Acid, Monohydroxy_Lipid	(R)-3-hydroxybutyrylcarnitine_Fatty Acid Metabolism (Acyl Carnitine, Hydroxy)_Lipid	X - 11847_NA_NA
43	N-acetylisoleucine_Leucine, Isoleucine and Valine Metabolism_Amino Acid	lidocaine_Drug - Analgesics, Anesthetics_Xenobiotics	1-linolenoylglycerol (18:3)_Monoacylglycerol_Lipid	5-acetylamino-6-formylamino-3-methyluracil_Xanthine Metabolism_Xenobiotics
44	uracil_Pyrimidine Metabolism, Uracil containing_Nucleotide	androstenediol (3beta,17beta) monosulfate (1)_Androgenic Steroids_Lipid	erucate (22:1n9)_Long Chain Monounsaturated Fatty Acid_Lipid	5-dodecenoate (12:1n7)_Medium Chain Fatty Acid_Lipid
45	glycolithocholate_Secondary Bile Acid Metabolism_Lipid	arachidonoylcholine_Fatty Acid Metabolism (Acyl Choline)_Lipid	oleoylcholine_Fatty Acid Metabolism (Acyl Choline)_Lipid	biliverdin_Hemoglobin and Porphyrin Metabolism_Cofactors and Vitamins
46	X - 12127_NA_NA	3-hydroxy-2-methylpyridine sulfate_Chemical_Xenobiotics	X - 17328_NA_NA	stearidonate (18:4n3)_Long Chain Polyunsaturated Fatty Acid (n3 and n6)_Lipid
47	androstenediol (3beta,17beta) monosulfate (2)_Androgenic Steroids_Lipid	sucrose_Disaccharides and Oligosaccharides_Carbohydrate	X - 16946_NA_NA	cystathionine_Methionine, Cysteine, SAM and Taurine Metabolism_Amino Acid
48	1-myristoylglycerol (14:0)_Monoacylglycerol_Lipid	N6-carboxymethyllysine_Advanced Glycation End-product_Carbohydrate	pipecolate_Lysine Metabolism_Amino Acid	pyridoxate_Vitamin B6 Metabolism_Cofactors and Vitamins
49	methyl-4-hydroxybenzoate sulfate_Benzoate Metabolism_Xenobiotics	X - 23644_NA_NA	S-methylmethionine_Methionine, Cysteine, SAM and Taurine Metabolism_Amino Acid	tauro-beta-muricholate_Primary Bile Acid Metabolism_Lipid
50	laurate (12:0)_Medium Chain Fatty Acid_Lipid	taurodeoxycholate_Secondary Bile Acid Metabolism_Lipid	X - 16576_NA_NA	butyrylcarnitine (C4)_Fatty Acid Metabolism (also BCAA Metabolism)_Lipid

51	5alpha-androstan-3beta,17beta-diol disulfate_Androgenic Steroids_Lipid	inosine_Purine Metabolism, (Hypo)Xanthine/Inosine containing_Nucleotide	4-allylcatechol sulfate_Benzoate Metabolism_Xenobiotics	X - 16124_NA_NA

Table S3. Metabolites chosen in each of the final machine learning models.

	lgbm (Light GBM)	lg (Logistic regression)	rf (Random forests)
0	X _ 11308_NA_NA	X _ 24970_NA_NA	X _ 11308_NA_NA
1	X _ 24970_NA_NA	X _ 11372_NA_NA	perfluorooctanoate _PFOA__Chemical_Xenobiotics
2	perfluorooctanoate _PFOA__Chemical_Xenobiotics	X _ 11308_NA_NA	N_acetyl_2_aminooctanoate__Fatty Acid_ Amino_Lipid
3	X _ 24307_NA_NA	X _ 14939_NA_NA	X _ 24970_NA_NA
4	X _ 11372_NA_NA	fructose_Fructose_ Mannose and Galactose Metabolism_Carbohydrate	N6_methyllysine_Lysine Metabolism_Amino Acid
5	X _ 17653_NA_NA	1__1_enyl_palmitoyl__GPC _P_16_0___Lysoplasmalogen_Lipid	X _ 23636_NA_NA
6	X _ 12112_NA_NA	ferulic acid 4_sulfate_Food Component_Plant_Xenobiotics	methionine sulfone_Methionine_ Cysteine_ SAM and Taurine Metabolism_Amino Acid
7		ribulonate_xylulonate_lyxonate__Pentose Metabolism_Carbohydrate	
8		X _ 17676_NA_NA	
9		X _ 12680_NA_NA	
10		4_vinylguaiacol sulfate_Food Component_Plant_Xenobiotics	
11		X _ 11444_NA_NA	
12		X _ 24432_NA_NA	
13		3_hydroxystachydrine__Food Component_Plant_Xenobiotics	
14		1_palmitoyl_2_palmitoleoyl_GPC _16_0_16_1___Phosphatidylcholine _PC__Lipid	
15		ethyl alpha_glucopyranoside_Food Component_Plant_Xenobiotics	
16		1_2_dilinoleoyl_GPC _18_2_18_2__Phosphatidylcholine _PC__Lipid	
17		metabolonic lactone sulfate_Partially Characterized Molecules_Partially Characterized Molecules	
18		X _ 24337_NA_NA	
19		X _ 21353_NA_NA	
20		behenoyl sphingomyelin _d18_1_22_0___Sphingomyelins_Lipid	
21		1__1_enyl_palmitoyl__2_linoleoyl_GPC _P_16_0_18_2___Plasmalogen_Lipid	
22		sphingomyelin _d18_1_22_1_ d18_2_22_0_ d16_1_24_1___Sphingomyelins_Lipid	
23		N_methylpipecolate_Bacterial_Fungal_Xenobiotics	
24		1_lignoceroyl_GPC _24_0__Lysophospholipid_Lipid	
25		X _ 21448_NA_NA	

26		branched_chain_ straight_chain_ or cyclopropyl 10_1 fatty acid _1___Partially Characterized Molecules_Partially Characterized Molecules	
27		1__1_enyl_palmitoyl__2_palmitoyl_GPC _P_16_0_16_0___Plasmalogen_Lipid	
28		X _ 12524_NA_NA	
29		octadecenedioylcarnitine _C18_1_DC___Fatty Acid Metabolism _Acyl Carnitine_ Dicarboxylate__Lipid	
30		1_stearoyl_2_oleoyl_GPI _18_0_18_1___Phosphatidylinositol _PI__Lipid	
31		creatinine_Creatine Metabolism_Amino Acid	
32		sphingomyelin _d18_1_21_0_ d17_1_22_0_ d16_1_23_0___Sphingomyelins_Lipid	
33		X _ 12112_NA_NA	
34		X _ 13728_NA_NA	
35		X _ 23636_NA_NA	
36		X _ 21410_NA_NA	
37		4_methoxyphenol sulfate_Tyrosine Metabolism_Amino Acid	
38		X _ 18922_NA_NA	
39		X _ 16397_NA_NA	
40		alpha_ketobutyrate_Methionine_ Cysteine_ SAM and Taurine Metabolism_Amino Acid	
41		hypotaurine_Methionine_ Cysteine_ SAM and Taurine Metabolism_Amino Acid	
42		2_hydroxybutyrate_2_hydroxyisobutyrate_Glutathione Metabolism_Amino Acid	
43		3_methylglutaconate_Leucine_ Isoleucine and Valine Metabolism_Amino Acid	
44		glucuronide of C12H22O4 _1___Partially Characterized Molecules_Partially Characterized Molecules	
45		octadecenedioate _C18_1_DC__Fatty Acid_ Dicarboxylate_Lipid	
46		octadecadienedioate _C18_2_DC___Fatty Acid_ Dicarboxylate_Lipid	
47		1_stearoyl_2_linoleoyl_GPC _18_0_18_2___Phosphatidylcholine _PC__Lipid	
48		myristoleoylcarnitine _C14_1___Fatty Acid Metabolism _Acyl Carnitine_ Monounsaturated__Lipid	
49		X _ 14056_NA_NA	
50		X _ 12729_NA_NA	
51		1_oleoyl_GPC _18_1__Lysophospholipid_Lipid	
52		1_ribosyl_imidazoleacetate__Histidine Metabolism_Amino Acid	
53		1_dihomo_linolenylglycerol _20_3__Monoacylglycerol_Lipid	
54		cysteinylglycine disulfide__Glutathione Metabolism_Amino Acid	
55		X _ 12100_NA_NA	

56		arabonate_xylonate_Pentose Metabolism_Carbohydrate	
57		glycine_Glycine_ Serine and Threonine Metabolism_Amino Acid	
58		1__1_enyl_palmitoyl__2_palmitoleoyl_GPC _P_16_0_16_1___Plasmalogen_Lipid	
59		X _ 21383_NA_NA	
60		glycerophosphoglycerol_Glycerolipid Metabolism_Lipid	
61		1_palmitoleoylglycerol _16_1___Monoacylglycerol_Lipid	
62		N1_methylinosine_Purine Metabolism_ _Hypo_Xanthine_Inosine containing_Nucleotide	
63		X _ 11880_NA_NA	
64		X _ 11470_NA_NA	
65		X _ 21319_NA_NA	
66		X _ 24947_NA_NA	
67		X _ 12906_NA_NA	
68		sphingomyelin _d18_1_17_0_ d17_1_18_0_ d19_1_16_0__Sphingomyelins_Lipid	
69		X _ 12104_NA_NA	
70		1__1_enyl_stearoyl__2_oleoyl_GPE _P_18_0_18_1__Plasmalogen_Lipid	
71		X _ 23665_NA_NA	
72		X _ 22162_NA_NA	
73		X _ 13553_NA_NA	
74		heptenedioate _C7_1_DC___Fatty Acid_ Dicarboxylate_Lipid	

Table S4. Metabolites chosen in each of the final machine learning models without xenobiotics.

	lgbm (Light GBM)	lg (Logistic regression)	rf (Random forests)
0	X _ 11308_NA_NA	X _ 11308_NA_NA	X _ 11308_NA_NA
1	X _ 24970_NA_NA	X _ 11372_NA_NA	3_carboxy_4_methyl_5_propyl_2_furanpropanoate _CMPF__Fatty Acid_ Dicarboxylate_Lipid
2	X _ 24307_NA_NA	X _ 24970_NA_NA	X _ 24970_NA_NA
3	gulonate__Ascorbate and Aldarate Metabolism_Cofactors and Vitamins	X _ 16935_NA_NA	N6_N6_dimethyllysine_Lysine Metabolism_Amino Acid
4	N_acetyltaurine_Methionine_ Cysteine_ SAM and Taurine Metabolism_Amino Acid	X _ 11880_NA_NA	bilirubin_Hemoglobin and Porphyrin Metabolism_Cofactors and Vitamins
5	fructose_Fructose_ Mannose and Galactose Metabolism_Carbohydrate	N6_methyllysine_Lysine Metabolism_Amino Acid	sphingomyelin _d18_1_20_1_ d18_2_20_0___Sphingomyelins_Lipid
6	arabonate_xylonate_Pentose Metabolism_Carbohydrate	phenylpyruvate_Phenylalanine Metabolism_Amino Acid	5_methyluridine _ribothymidine__Pyrimidine Metabolism_ Uracil containing_Nucleotide
7	1_oleoyl_GPC _18_1__Lysophospholipid_Lipid	gamma_glutamyl_epsilon_lysine_Gamma_glutamyl Amino Acid_Peptide	
8	X _ 13431_NA_NA	hydroxy_CMPF__Fatty Acid_ Dicarboxylate_Lipid	
9	X _ 12112_NA_NA	behenoyl sphingomyelin _d18_1_22_0___Sphingomyelins_Lipid	
10	X _ 12100_NA_NA	2_palmitoyl_GPC_ _16_0___Lysophospholipid_Lipid	
11	X _ 18921_NA_NA	X _ 23666_NA_NA	
12	X _ 12104_NA_NA	N6_N6_dimethyllysine_Lysine Metabolism_Amino Acid	
13	X _ 17676_NA_NA	sphingomyelin _d18_1_22_1_ d18_2_22_0_ d16_1_24_1___Sphingomyelins_Lipid	
14	mannose_Fructose_ Mannose and Galactose Metabolism_Carbohydrate	X _ 11441_NA_NA	
15	X _ 15503_NA_NA	methionine sulfone_Methionine_ Cysteine_ SAM and Taurine Metabolism_Amino Acid	
16	X _ 12193_NA_NA	X _ 15245_NA_NA	
17	X _ 21829_NA_NA	retinol _Vitamin A__Vitamin A Metabolism_Cofactors and Vitamins	
18	2_palmitoleoyl_GPC_ _16_1___Lysophospholipid_Lipid	1_linoleoyl_GPG _18_2___Lysophospholipid_Lipid	
19	9_hydroxystearate_Fatty Acid_ Monohydroxy_Lipid	X _ 15503_NA_NA	
20	sphingomyelin _d18_1_24_1_ d18_2_24_0___Sphingomyelins_Lipid	glutamate_Glutamate Metabolism_Amino Acid	
21	sphingomyelin _d18_2_14_0_ d18_1_14_1___Sphingomyelins_Lipid	dehydroepiandrosterone sulfate _DHEA_S__Androgenic Steroids_Lipid	
22	X _ 18887_NA_NA	4_hydroxyglutamate_Glutamate Metabolism_Amino Acid	
23	X _ 22162_NA_NA	androstenediol _3alpha_ 17alpha_ monosulfate _3__Androgenic Steroids_Lipid	
24	X _ 12680_NA_NA	X _ 12524_NA_NA	
25	X _ 12101_NA_NA	X _ 21364_NA_NA	
26	myristoleoylcarnitine _C14_1___Fatty Acid Metabolism _Acyl Carnitine_ Monounsaturated__Lipid	3_amino_2_piperidone_Urea cycle_ Arginine and Proline Metabolism_Amino Acid	

27	X _ 22771_NA_NA	andro steroid monosulfate C19H28O6S _1___Androgenic Steroids_Lipid	
28	1__1_enyl_palmitoyl__2_palmitoyl_GPC _P_16_0_16_0___Plasmalogen_Lipid	taurochenodeoxycholic acid 3_sulfate_Secondary Bile Acid Metabolism_Lipid	
29	X _ 19141_NA_NA	sphingomyelin _d18_1_20_0_ d16_1_22_0___Sphingomyelins_Lipid	
30	X _ 21467_NA_NA	3__4_hydroxyphenyl_lactate _HPLA__Tyrosine Metabolism_Amino Acid	
31	X _ 21470_NA_NA	tricosanoyl sphingomyelin _d18_1_23_0___Sphingomyelins_Lipid	
32	X _ 21471_NA_NA	isovalerate _C5__Leucine_ Isoleucine and Valine Metabolism_Amino Acid	
33	alpha_hydroxyisovalerate_Leucine_ Isoleucine and Valine Metabolism_Amino Acid	sphingomyelin _d18_2_23_0_ d18_1_23_1_ d17_1_24_1___Sphingomyelins_Lipid	
34	N_acetylglucosamine_N_acetylgalactosamine_Aminosugar Metabolism_Carbohydrate	dihomo_linoleoylcarnitine _C20_2___Fatty Acid Metabolism _Acyl Carnitine_ Polyunsaturated__Lipid	
35	3_methylglutarylcarnitine _2__Leucine_ Isoleucine and Valine Metabolism_Amino Acid	X _ 14939_NA_NA	
36	X _ 11299_NA_NA	sphingomyelin _d17_2_16_0_ d18_2_15_0___Sphingomyelins_Lipid	
37	X _ 11372_NA_NA	tetradecanedioate _C14__Fatty Acid_ Dicarboxylate_Lipid	
38	X _ 11470_NA_NA	N2_N2_dimethylguanosine_Purine Metabolism_ Guanine containing_Nucleotide	
39	X _ 11478_NA_NA	1_linoleoyl_2_arachidonoyl_GPC _18_2_20_4n6___Phosphatidylcholine _PC__Lipid	
40	X _ 17654_NA_NA	1_palmitoyl_2_oleoyl_GPC _16_0_18_1__Phosphatidylcholine _PC__Lipid	
41	X _ 21736_NA_NA	1__1_enyl_palmitoyl__2_oleoyl_GPE _P_16_0_18_1___Plasmalogen_Lipid	
42	1_dihomo_linolenylglycerol _20_3__Monoacylglycerol_Lipid	ceramide _d18_1_14_0_ d16_1_16_0___Ceramides_Lipid	
43	X _ 17351_NA_NA	X _ 23997_NA_NA	
44	X _ 16397_NA_NA	1__1_enyl_palmitoyl__GPC _P_16_0___Lysoplasmalogen_Lipid	
45	X _ 16580_NA_NA	2_oleoylglycerol _18_1__Monoacylglycerol_Lipid	
46	bilirubin _E_Z or Z_E___Hemoglobin and Porphyrin Metabolism_Cofactors and Vitamins	guanidinoacetate_Creatine Metabolism_Amino Acid	
47	X _ 12026_NA_NA	N1_methylinosine_Purine Metabolism_ _Hypo_Xanthine_Inosine containing_Nucleotide	
48	X _ 12221_NA_NA	2_hydroxy_3_methylvalerate_Leucine_ Isoleucine and Valine Metabolism_Amino Acid	
49	X _ 12906_NA_NA	X _ 21448_NA_NA	
50	X _ 13553_NA_NA	4_hydroxy_2_oxoglutaric acid_Fatty Acid_ Dicarboxylate_Lipid	

51	X _ 17357_NA_NA	3_methoxytyrosine_Tyrosine Metabolism_Amino Acid	
52	X _ 18899_NA_NA	urate_Purine Metabolism_ _Hypo_Xanthine_Inosine containing_Nucleotide	
53	1__1_enyl_stearoyl__2_linoleoyl_GPE _P_18_0_18_2___Plasmalogen_Lipid	sphingomyelin _d18_0_18_0_ d19_0_17_0___Dihydrosphingomyelins_Lipid	
54	X _ 21448_NA_NA	palmitoyl sphingomyelin _d18_1_16_0__Sphingomyelins_Lipid	
55	cysteinylglycine disulfide__Glutathione Metabolism_Amino Acid	tiglyl carnitine _C5__Leucine_ Isoleucine and Valine Metabolism_Amino Acid	
56	X _ 24947_NA_NA	1_arachidonoyl_GPI_ _20_4___Lysophospholipid_Lipid	
57	X _ 24951_NA_NA	1__1_enyl_stearoyl__2_linoleoyl_GPE _P_18_0_18_2___Plasmalogen_Lipid	
58	uridine_Pyrimidine Metabolism_ Uracil containing_Nucleotide	5_hydroxylysine_Lysine Metabolism_Amino Acid	
59	nisinate _24_6n3__Long Chain Polyunsaturated Fatty Acid _n3 and n6__Lipid	N_acetylneuraminate_Aminosugar Metabolism_Carbohydrate	
60	_N_1_ _ N_8___acetylspermidine_Polyamine Metabolism_Amino Acid	isoleucine_Leucine_ Isoleucine and Valine Metabolism_Amino Acid	
61	glycine_Glycine_ Serine and Threonine Metabolism_Amino Acid	X _ 21471_NA_NA	
62	histidine_Histidine Metabolism_Amino Acid	kynurenate_Tryptophan Metabolism_Amino Acid	
63	hypotaurine_Methionine_ Cysteine_ SAM and Taurine Metabolism_Amino Acid	X _ 24947_NA_NA	
64	leucine_Leucine_ Isoleucine and Valine Metabolism_Amino Acid	palmitoyl_sphingosine_phosphoethanolamine _d18_1_16_0__Ceramide PEs_Lipid	
65	dihydroorotate_Pyrimidine Metabolism_ Orotate containing_Nucleotide	linoleoylcarnitine _C18_2___Fatty Acid Metabolism _Acyl Carnitine_ Polyunsaturated__Lipid	
66	gamma_glutamylcitrulline__Gamma_glutamyl Amino Acid_Peptide	sphingomyelin _d18_2_24_2___Sphingomyelins_Lipid	
67	N_oleoylserine_Endocannabinoid_Lipid	N_oleoylserine_Endocannabinoid_Lipid	
68	glycine conjugate of C10H14O2 _1___Partially Characterized Molecules_Partially Characterized Molecules	1_linolenoyl_GPC _18_3___Lysophospholipid_Lipid	
